# Integration of Screening and Referral Tools for Social Determinants of Health and Modifiable Lifestyle Factors in the Epic Electronic Health Record System: Scoping Review

**DOI:** 10.2196/73615

**Published:** 2025-09-15

**Authors:** Jawad Ahmed Chishtie, Jenice Tea, Manuel Ester, Gehna Rasheed, Nicelle Chua, Marcus Vaska, Gary Teare, Kamala Adhikari

**Affiliations:** 1 Cancer Prevention and Screening Innovation Public Health Evidence and Innovation Primary Care Alberta Calgary, AB Canada; 2 Department of Family Medicine Cumming School of Medicine University of Calgary Calgary, AB Canada; 3 Department of Community Health Sciences Cumming School of Medicine University of Calgary Calgary, AB Canada; 4 McCaig Institute for Bone and Joint Health Cumming School of Medicine University of Calgary Calgary, AB Canada; 5 O’Brien Institute for Public Health Cumming School of Medicine University of Calgary Calgary, AB Canada; 6 Library Services Acute Care Alberta Calgary, AB Canada

**Keywords:** electronic health record, Epic EHR system, social determinants of health, modifiable risk factors, integration, optimization.

## Abstract

**Background:**

Recent health behavior interventions combine social determinants of health (SDOH) and biosocial perspectives, refocusing from the individual to broader societal contexts under the SDOH approach. Targeting modifiable health behaviors can significantly reduce disease risk and save up to 30% of health care costs. Screening tools individual and societal factors are being increasingly integrated into electronic health record (EHR) systems. Epic Systems is a leading, most adopted EHRs worldwide, with modules on SDOH and modifiable risk factors. Literature on integration and use of screening tools for SDOH and modifiable risk factors is lacking.

**Objective:**

This review aimed to (1) summarize evidence integrating screening and referral tools for SDOH and modifiable risk factors including tobacco/alcohol use and physical inactivity in the Epic EHR; (2) synthesize findings on implementation methods, processes, clinical workflow modifications, and outcomes from integrating SDOH screening and referral tools in EHR systems; and (3) capture the major barriers, facilitators, and lessons learned across the included implementation studies.

**Methods:**

We followed Joanna Briggs Institute’s guidelines, Arksey and O’Malley’s framework, and the PRISMA-ScR (Preferred Reporting Items for Systematic Reviews and Meta-Analyses extension for Scoping Reviews) checklist. We included 3 peer-reviewed databases, 2 gray literature sources, and citation chaining from related reviews and articles.

**Results:**

All included studies (n=43) were from 24 US states; 26 reported quantitative methods, 12 reported mixed methods, and 6 were qualitative studies across various health settings. Most studies focused on adults, with the top 3 SDOH domains being housing, food and transportation, while physical activity, alcohol and tobacco were the most common modifiable risk factors. The top 3 SDOH domains were housing, food, and transportation, while physical activity, alcohol, and tobacco use were the most common risk factors targeted. Various screening tools were used, with the Protocol for Responding to & Assessing Patients’ Assets, Risks, and Experiences (PRAPARE) being used the most across 6 studies. Most integrations used enhanced support or optimized workflows, with MyChart and Best Practice Advisories being the most used Epic modules and functions. MyChart was the most patient-accepted module. Screening and referral patient outcomes varied, with many studies presenting a significant impact. The most important integration facilitators included leadership support, dedicated clinical champions, and well-defined roles; barriers included clinician time, inefficient workflows, and the availability of devices and staff to ensure integrated tools’ usage.

**Conclusions:**

Integration of SDOH and modifiable risk factors in the Epic EHR is being increasingly adopted to capture and target equitable health services. While Epic is among the most globally adopted EHRs, studies are primarily from the United States. Epic’s SDOH wheel module is insufficient in capturing context-based SDOH and behavioral domains. Need for contextual standardization of SDOH and modifiable risk factor domains and EHR tools is being increasingly felt. Future research is needed for enhanced learning, improvement and use of built-in and customized tools, standardization, and processes for integrating targeted patient-centered interventions.

## Introduction

### Background

The SDOH “approach” brings together the social determinants of health (SDOH), such as income, education, and social inclusion, with biosocial approaches to the health behavior dynamics, contextualizing individuals within their environments [1-3]. This shifts the focus from the individual to the broader encompassing structures and ideologies that influence health behaviors and inequities [3]. Among the varied health behaviors, modifiable risk factors, such as smoking, alcohol intake, and physical activity, are behaviors that can be changed to improve health and reduce risk of disease, and which account for up to 30% of health care costs in Canada [4]. Hence, there has been an increased focus on public health interventions targeting modifiable risk factors, particularly for preventing chronic noncommunicable disease [5].

While electronic health record (EHR) systems have improved quality of health care delivery [1,6], the current global focus is on maximizing their value toward continuous quality improvement and targeting inequalities within health care systems [1,7]. Integrating standardized screening and documentation in EHRs can transform providers’ ability to monitor risks, adjust care plans, and address unmet needs [8]. Such EHR tools allow screening for SDOH and behavioral risk factors for addressing unmet social needs, and improve connections to relevant resources [9].

### Rationale

The main objective of this review is to summarize evidence on the integration of screening and referral tools for SDOH and modifiable risk factors, including tobacco use, alcohol use, and physical inactivity, in the Epic EHR. We further synthesize findings on implementation methods, processes, modifications to clinical workflows, and outcomes from integrating SDOH screening and referral tools in EHR systems. Finally, a third objective is to capture the major barriers, facilitators, and lessons learned across the included implementation studies.

Epic Systems [10] (Epic Systems Corporation) is an early innovator and among the 5 leading and most globally adopted EHRs [11]. In 2023, Epic was in use at 89% of acute care hospitals in the United States [11]. Recently, Alberta Health Services (AHS), Alberta, Canada, transitioned to a unified Epic-based system called Connect Care, sparking great interest for the use of the EHR as a unified, single source of truth and an integrated care tool [12].

We identified related recent reviews that directly or indirectly included the use of EHRs to capture SDOH and health behaviors, with particular focus on the United States. Rangachari and Thapa’s [13] systematic review evaluated the effectiveness of hospital-based and system initiatives for SDOH integrations in the United States. Li et al [14] and Ganatra et al [15] summarized gaps in the standardized collection of SDOH data. Caicedo and colleagues’ [16] review focused on patient perspectives and experiences of integrating SDOH information in primary care EHRs using qualitative data. Yan and colleagues’ [17] systematic review summarized the effectiveness of social needs screening and interventions in clinical settings from the United States [17]. Bompelli and colleagues’ [18] review synthesized machine learning approaches for studying SDOH domains, while Cook et al [19] highlighted issues related to quality and bias of EHR SDOH data. Thoele et al [20] summarized strategies for promoting screening, brief intervention, and referral (SBIR) in health care settings. Chen et al [1] summarized impacts on risk prediction, including health service utilization among patients after integrating SDOH domains in EHRs. Venzon and colleagues’ [21] review summarized capturing SDOH domains in electronic systems, including surveys and EHRs, in the United States.

As can be inferred, most reviews have not examined strategies for integrating SDOH and modifiable risk factor screening, support, and referral in clinical care using the Epic EHR. In addition, existing reviews do not present findings on specific EHRs, related challenges, lessons, processes, and approaches involved in integrating screening and referral tools, and are mostly limited to findings from the United States. We attempt to fill this gap in literature by capturing global peer-reviewed and gray literature, focusing on the Epic EHR system, as one of the largest and globally adopted EHR systems.

## Methods

### Scoping Review Methods

We used established scoping review methods to summarize the extent and characteristics of evidence on the use of screening tools for SDOH and modifiable risk factors in the Epic EHR [22,23]. Based on Joanna Briggs Institute’s guidance [24] and Arksey and O’Malley’s seminal framework [25], we incorporated improvements suggested by Levac et al [26] and Peters et al [27]. A brief protocol was developed and peer-reviewed internally by subject matter experts in 3 rounds. In the first round, 12 seminal articles were reviewed to develop the scope of this review. This was presented to our multidisciplinary team, where further information on the current state, usability of the review, and value-add was discussed. The protocol was expanded to address feedback from the team, while an information specialist was consulted for developing the search strategy. Seminal papers were also shared with the information specialist. The second iteration of the protocol included the search planning document (see Multimedia Appendix 1), along with the rationale detailing the current literature on the subject. In the third round, 2 senior scientists reviewed the protocol and suggested improvements. The protocol was not published. The PRISMA-ScR (Preferred Reporting Items for Systematic Reviews and Meta-Analyses extension for Scoping Reviews) checklist is used to report our findings [28] (see Multimedia Appendix 2).

### Eligibility Criteria

We used the (population, intervention, comparison, outcome, and study type) PICOS (population, intervention, comparison, outcome, and study type) criteria to conceptualize the search strategy and the eligibility criteria for article selection [24], presented in Textbox 1. Briefly, articles in English were included if they presented results from any intervention aimed at integrating screening and referral tools for SDOH or behavioral risk factors within the Epic EHR. No restrictions were placed on the population, time, or outcome. We included randomized and nonrandomized implementation trials, longitudinal and cross-sectional observational studies, and qualitative studies. Articles were excluded if they did not include the intervention of interest, did not use the Epic EHR, or were classified as editorials, short papers, conference abstracts, or reviews.

Textbox 2 shows the eligibility criteria.

**Population, intervention, comparison, outcome, and study type (PICOS) criteria for study eligibility.**

- Population: All populations.
- Intervention: Integration of tools, modules, documentation, alerts, or other assistive workflows to screen for social determinants of health (SDOH) and lifestyle risk factors (tobacco, alcohol, and physical inactivity) using the Epic electronic health record (EHR), including referrals and other support to address patient needs after screening.
- Comparison: Not applicable.
- Outcome: Quantitative or qualitative outcomes on processes, experiences, clinical or health service outcomes.
- Study type: Randomized and nonrandomized implementation trials, longitudinal and cross-sectional observational studies, qualitative reports, and gray literature reports.

**Eligibility criteria for the selection of articles.**

Inclusion criteria:

- Article type: Peer-reviewed or conference papers.
- Language: English.
- Date range: Any date till January 2024.
- Study designs: Quantitative, qualitative, or mixed methods designs.
- Electronic health record (EHR): Epic EHR only.
- EHR integrations: Articles related to social determinants of health (SDOH) or modifiable risk factors, including tobacco (smoking), alcohol, and physical inactivity.
- EHR Implementation and evaluation types: Epic EHR-related tools, modules, documentation, alerts, assistive workflows.

Exclusion criteria:

- Article type: Editorials, commentaries, reviews, book chapters, short papers, or reports.
- Language: Articles not in English.
- Date range: Articles published after January 2024.
- Population: Not applicable.
- Study designs: Articles that do not mention a study design or are short reports.
- EHR: Any other EHR, such as Cerner.
- EHR integrations: Articles related to factors that are not classified within SDOH and modifiable risk factors.
- EHR implementation and evaluation types: Articles not mentioning details on Epic-related tools and workflows.

### Search Strategy

The search strategy was developed in 3 stages. A total of 2 researchers independently completed a preliminary search for seminal articles using Google Scholar, identifying 20 relevant articles based on the search terms: screening, SDOH, modifiable risk factors, alcohol, smoking, physical activity, and Epic EHR. These seminal articles were used to develop the search concepts, build search terms, define the eligibility criteria, and sources of evidence. This was shared with the information specialist, who then developed the detailed search strategy, using the 3 main concepts of: screening or referral, SDOH or behavioral risk factors, and Epic EHR. The search strategy was peer-reviewed by the multidisciplinary team and finalized with the information specialist. The MEDLINE search strategy is presented in Multimedia Appendix 3, while the complete search planning document for all platforms is presented in Multimedia Appendix 1. There was no time limit placed on the search, and all sources were searched till January 08, 2024. A search for gray literature was conducted using the same concepts and combination of keywords.

### Sources of Information

Systematic database searches included MEDLINE, Embase, PubMed, and CINAHL, while gray literature sources were Google, Google Scholar, Epic UserWeb, and Epic research sites. The first 400 results were screened from each gray literature source. Citation chaining was undertaken from seminal articles.

### Selection of Sources of Evidence

References were imported into Covidence [29] for managing the screening process. Deduplication was performed in Covidence, comparing titles, authors, and abstracts of the papers included. Title and abstract screening were undertaken by 2 independent reviewers. All reviewers met twice to resolve conflicts during this phase. A total of 4 reviewers independently assessed articles during full text screening, while conflicts at each stage were arbitrated by another expert reviewer.

### Data Charting Process

A data abstraction spreadsheet was developed in Microsoft Excel and piloted on 8 articles. To minimize bias, reviewers met weekly to refine the abstraction process, maintain consistency, and discuss emerging findings. As this was a scoping review, with varied results from peer-reviewed and gray literature, we did not conduct a formal risk of bias assessment.

### Data Items

Abstracted data included author, title, journal, publication date, locations, study objectives, study characteristics of interest, SDOH or behavioral factor domains, intervention and implementation strategies, EHR tools, processes for integration of EHR tools, evaluation data sources, and quantitative and qualitative findings.

### Synthesis of Results

Qualitative findings included lessons learned, facilitators, and barriers experienced in the studies. Quantitative findings included screening rates, referral rates, the rate of patients with a positive SDOH screen, and SDOH factors that were most identified in patients. For each article, data abstraction was conducted in 2 iterations, first completed by a researcher, and then validated by an independent expert researcher.

To better understand the potential impact of the different approaches to implementing SDOH and behavioral risk factor screening and referral tools within the EHR, we calculated summary statistics for reported screening and referral rates, referral to paper-based or web-based resources, referral to another provider, and refused referral by the patient. We used pooled results from studies reporting these aspects, following the Joanna Briggs Institute’s guidance on meta-analysis [30]. We aggregated the mean (SD) across studies.

Barriers, facilitators, and lessons were synthesized across studies using an inductive approach using qualitative content analysis [31]. A coding structure was established, with items grouped according to common themes by a researcher. This was followed by refinement of themes by the team to ensure themes reflected the captured data.

## Results

### Selection of Sources of Evidence

**Figure 1:**
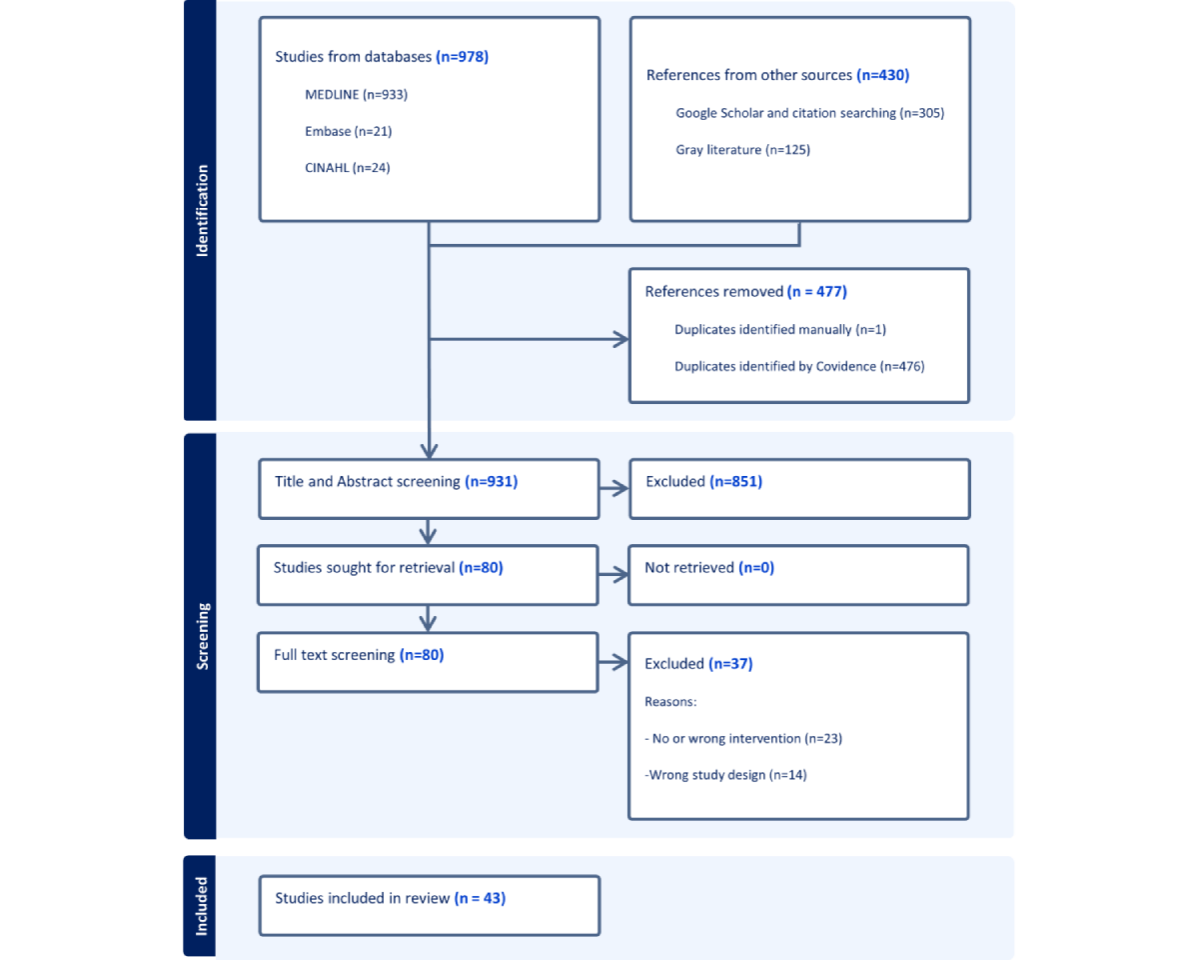
Preferred Reporting Items for Systematic Reviews and Meta-Analyses (PRISMA) flow diagram detailing study search results and screening process.

The search yielded 1408 articles, with 931 articles remaining after de-deduplication that included 933 articles from Ovid MEDLINE, 21 from Embase, 24 from CINAHL, 305 from Google Scholar, and citation chaining, and 125 from gray literature sources. During title and abstract screening, 851 articles were excluded as they did not meet the eligibility criteria, while 80 articles were sought for retrieval and full text screening. All articles were retrieved, while 37 were excluded after full-text screening, as these either did not have the right study design or intervention. Data from all 43 studies were charted and included for synthesis. Critical appraisal of individual sources of evidence was not conducted. Figure 1 presents the PRISMA flow chart. Study characteristics, author, year of publication, location, settings, population, study type and design, SDOH domains, intervention, and Epic tools are presented in Multimedia Appendix 4.

### Study Characteristics

#### Study Year, Location, Design, and Data Sources

All 43 articles were published between 2017 to 2024, with a third published in 2023 (14/43, 33%). An upper trend was observed from 2021 to 2023, with most literature from 2021 onwards (26/43, 60.5%).

**Figure 2:**
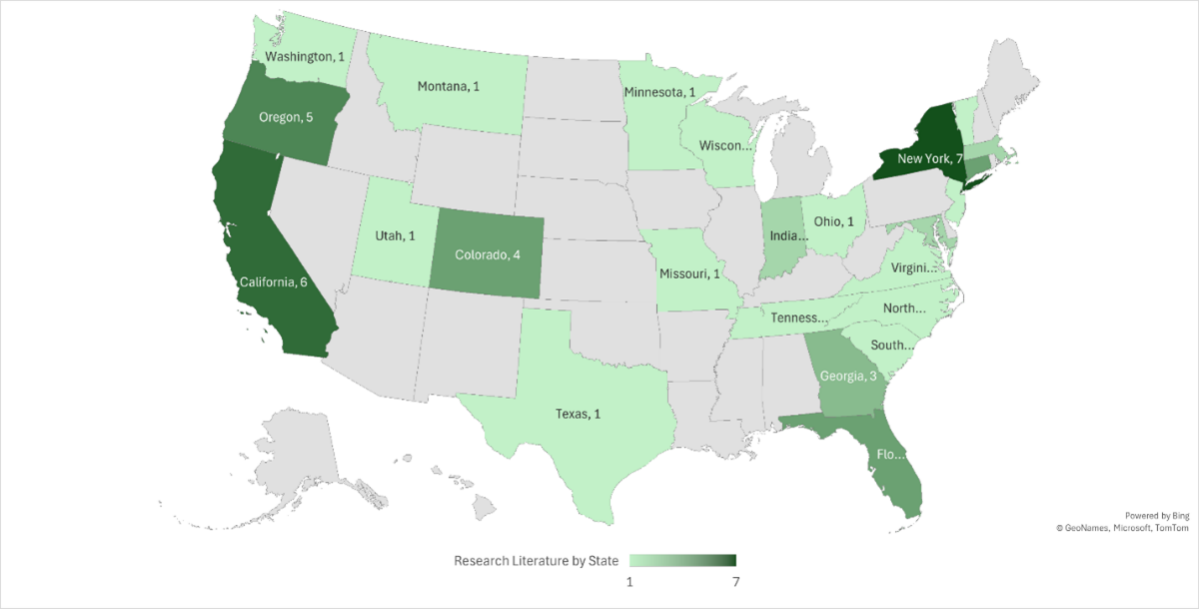
Research literature by state.

Table 1 details the objectives, settings, and designs. All studies were from the United States, specifically from 24 states, with the most common being New York (7/43, 16%), California (6/43, 14%), and Oregon (5/43, 12%). The rest included Colorado, Connecticut, Florida, Georgia, Indiana, Maryland, Massachusetts, Minnesota, Missouri, Montana, New Jersey, New York, North Carolina, Ohio, South Carolina, Tennessee, Texas, Utah, Vermont, Virginia, Washington, and Wisconsin. Figure 2 demonstrates the distribution of publications by state.

Among the 43 included studies, methods included quantitative (26/43, 60%), mixed methods (12/43, 28%), and qualitative (5/43, 12%) designs. Quantitative designs included retrospective cohort (16/43, 37%), experimental nonrandomized (10/43, 23%), cross-sectional (10/43, 23%), prospective cohort (5/43, 12%), and experimental randomized (2/43, 5%). Most studies used data directly from the Epic EHR (30/43, 70%), while others used EHR and staff data (7/43, 16%), EHR, staff, and patient data (4/43, 9%), staff and patient data (1/43, 2%), or staff data only (1/43, 2%).

**Table 1:** Objectives, social determinants of health (SDOH) domains covered, settings, and design of included studies.

Reference	Location	Objective	SDOH^a^ components	Setting	Study type	Design
Angah et al [32], 2024	Connecticut, United States	Increase standardized HRSN^b^ screening and collection	Food insecurity, transportation, financial strain, and housing insecurity	Primary care and inpatient departments	Quantitative	Prospective cohort
Barclay et al [33], 2019	North Carolina, United States	Describe the implementation of screening and counseling for unhealthy alcohol use	Alcohol use	General internal medicine clinic	Qualitative	Experimental (nonrandomized trial)
Berkowitz et al [34], 2021	California, United States	Screen and address SDOH, socioemotional development, and perinatal depression in pediatric practices	Financial strain, transportation, stress, depression, violence, social connections, physical activity, and alcohol	Ambulatory clinic	Quantitative	Experimental (nonrandomized trial)
Brennan et al [35], 2022	Indiana, The United States	Screen and address SDOH, socioemotional development, and perinatal depression	N/A^c^	Pediatric practices	Quantitative	Prospective cohort
Buitron de la Vega et al [9], 2019	Massachusetts, United States	Understand social needs and the feasibility of implementing SDOH screening	Housing insecurity, food insecurity, medication affordability, transportation, utilities, caregiving, employment, and education	General internal medicine clinic	Quantitative	Experimental (nonrandomized trial)
Bunce et al [36], 2023	Oregon, United States	Explore how patient SDOH information influences clinician decisions	N/A	Primary care clinics	Quantitative	Prospective cohort
Burdick et al [37], 2017	Vermont, United States	Implement and evaluate SBIR^d^ integration on clinical outcomes	Alcohol, depression, and substance abuse	Primary care practices	Quantitative	Retrospective cohort
Cottrell et al [38], 2019	United States (20 states)	Examine the adoption of SDOH screening	Sex, race and ethnicity, language, age, income, insurance status, homelessness, and migrant status	Community health centers	Quantitative	Retrospective cohort
Eakin et al [39], 2023	California, United States	Assess the effects of California State Bill 1152 on ED^e^ visits among the homeless	Housing insecurity	Stanford University Hospital	Mixed methods	Prospective cohort
Fiori et al [40], 2019	New York, United States	Develop and implement the community linkage to care program and illustrate SDOH screening	Housing insecurity and quality, food, health care, utilities, transportation, childcare, and violence	Ambulatory pediatric clinics	Mixed methods	Prospective cohort
Fiori et al [41], 2020	New York, United States	Assess integrating SDOH screening and referral in a primary care setting	Housing security, housing quality, benefits (such as utilities), food insecurity, transportation, medication, or health care access, childcare or older adult care assistance, legal services, relationship concerns, and safety issues	Urban ambulatory pediatric clinics	Quantitative	Prospective cohort
Friedman et al [42], 2018	Washington and Oregon, United States	Create and describe tools and processes to address SDOH	Social, economic, environmental, and health education	Medical offices and hospitals	Quantitative	Retrospective cohort
Garg et al [43], 2023	Massachusetts, United States	Assess the implementation and effectiveness of the WE CARE social care system for low-income children	Childcare, education, employment, food security, housing security, household heat, and language	Community health centers	Quantitative	Experimental (randomized trial)
Gold et al [44], 2018	Oregon, United States	Assess the feasibility of implementing EHR^f^ tools for collecting, reviewing, and acting on SDOH data	Alcohol, race, ethnicity, tobacco use or exposure, depression, education, financial resource strain, housing insecurity, food insecurity, exposure to violence, physical inactivity, social isolation, and stress	Community health centers	Mixed methods	Prospective cohort
Gold et al [8], 2023	The United States (8 states)	Examine improvement in EHR screening of social risks	Child or family care insecurity, education, employment, financial strain, food insecurity, health insurance, health literacy, housing instability, inadequate physical activity, relationship safety, social isolation, stress, transportation needs, and utilities insecurity	Community health center clinics	Quantitative	Cross-sectional
Gore et al [45], 2022	New York, United States	Implement a sustainable process to screen hospitalized adults to capture individuals who would benefit from social work and food insecurity resources	Food insecurity	Hospital	Quantitative	Experimental (nonrandomized trial)
Gray et al [46], 2023	Colorado, United States	Evaluate implementation of HRSN screening and potential expansion of SDOH screening	Housing insecurity, food insecurity, transportation, utilities, and safety	Pediatric primary care clinic	Quantitative	Cross-sectional
Grus et al [47], 2021	United States (5 states)	Understand facilitators for the integration of EHR-based SDOH screening	Financial resource strain, food insecurity, housing insecurity, relationship safety, inadequate physical activity, social connection or isolation, and stress	Community health centers	Qualitative	Experimental (randomized trial)
Gunn et al [48], 2023	United States (Multistate)	Evaluate the implementation of screening and community resource referral platforms (CRRPs) for social isolation and loneliness	Social isolation and loneliness	Community health centers	Mixed methods	Prospective cohort
Gupta et al [49], 2023	South Carolina, United States	Assess the feasibility and sustainability of SDOH screening and referrals	Food insecurity, housing instability, utility insecurity, transportation needs, financial instability, violence or abuse, language or educational needs, health literacy, social connectedness, and comorbidities	Private nonprofit health system, community health, inpatient case management, or ambulatory care and condition management programs	Quantitative	Prospective cohort
Hao et al [50], 2023	North Carolina, United States	Evaluate the feasibility and acceptability of implementing the SDOH screening instrument into routine clinical oncology practice	Alcohol use, tobacco use, financial strain, food insecurity, transportation, social connections, physical activity, stress, housing, depression, and intimate partner violence	American College of Surgeons Commission on Cancer-accredited cancer center (outpatient clinic)	Mixed methods	Prospective cohort
Hsu et al [51], 2018	Washington, United States	Describe the implementation of adding a lay health worker role into primary care and testing the feasibility and impact of the role	N/A	Primary care clinics	Mixed methods	Prospective cohort
Isaacs et al [52], 2022	North Carolina, United States	Introduce SDOH screening at clinics where there was no previous standard of care	Interpersonal violence, food security, financial strain, and transportation	Primary care clinic	Quantitative	Experimental (nonrandomized trial)
Jennings et al [53], 2022	Virginia, United States	Reduce health disparities in individuals with cystic fibrosis by screening and addressing SDOH	Housing insecurity, food insecurity, transportation, utilities, health-care access, medication access, income or employment, and education	Clinic	Quantitative	Experimental (nonrandomized trial)
Jose et al [54], 2020	Minnesota, United States	Enhance tobacco use treatment among cancer patients by an EHR-based automatic referral system	Tobacco use	Comprehensive cancer center (Mayo clinic)	Quantitative	Prospective cohort
Kepper et al [55], 2023	Missouri, United States	Understand factors and frequency of SDOH use in EHR for patients with prediabetes and diabetes	Employment, housing, and economic circumstances, education and literacy, social environment, primary support group, problems related to upbringing, and problems related to psychosocial circumstances	Academic medical center	Mixed methods	Cross-sectional
Khanna et al [56], 2021	Maryland, United States	Develop an implementation strategy for electronic referrals to the tobacco quitline	Tobacco use	Ambulatory clinics	Quantitative	Prospective cohort
Kostelanetz et al [57], 2022	Tennessee, United States	Evaluate provider perceptions for universal SDOH screening	Housing status, social support, financial strain, food insecurity, educational attainment, alcohol use, tobacco use, and drug use	Academic medical center	Mixed methods	Cross-sectional
Kroese et al [58], 2024	Virginia, United States	Identify food insecurity and provide resources in real time to pediatric patients	Food insecurity	Pediatric clinics, ED, and medical homes	Quantitative	Experimental (nonrandomized trial)
LeLaurin et al [59], 2023	Florida, United States	Assess parent perspectives on EHR-based social needs screening and documentation, and identify family-centered screening design and implementation	Physical activity, food insecurity, housing insecurity, transportation needs, caregiver education and work, caregiver health, child education, and safety and environment	Pediatric primary care clinics	Qualitative	Cross-sectional
LeLaurin [60], 2023	Florida, United States	Assess multilevel factors impacting EHR-based social needs intervention adoption	Not specified	Pediatric primary care clinics	Mixed methods	Cross-sectional
Lindenfeld et al [61], 2023	New York, United States	Identify system-level barriers to adopting SDOH screening	Living situation, food insecurity, transportation, utilities, and safety	Hospitals and community health centers	Quantitative	Retrospective cohort
McCarthy et al [62], 2021	New York, United States	Implement physical activity screening within an electronic kiosk check-in process for cardiology	Physical activity	Preventative cardiology clinic	Quantitative	Cross-sectional
McNeely et al [63], 2021	New York and Massachusetts, United States	Evaluate EHR screening for substance use in primary care	Alcohol and drug use	Primary care clinics	Quantitative	Experimental (nonrandomized trial)
Palacio et al [64], 2018	Florida, United States	Explore factors for integrating SDOH in a large, diverse health care system EHR	Physical activity, stress, housing insecurity, social connections, and medical access	Hospitals and outpatient facilities	Qualitative	Cross-sectional
Penedo et al [65], 2022	Florida, United States	Describe the feasibility and implementation of an EHR-integrated symptom and needs screening and referral system	Financial concerns, childcare, stress management, nutritional needs, emotional needs, and transportation	Ambulatory gynecology-oncology clinics	Mixed methods	Cross-sectional
Peretz et al [66], 2023	New York, United States	Describe the experience of implementing systemwide planning and process for SDOH screening	Housing insecurity, food insecurity, and transportation	ED	Qualitative	Experimental (nonrandomized trial)
Rogers et al [67], 2022	New Jersey, United States	Describe the implementation strategy to identify and screen inpatients, outpatients, and ED patients for SDOH using EHR	Housing instability, food insecurity, transportation problems, utilities, and interpersonal safety	Hospital	Quantitative	Prospective cohort
Rudisill et al [68], 2023	South Carolina, United States	Identify barriers and facilitators of SDOH screening in primary care to inform future screening	Food insecurity, financial strain, lack of transportation, stress, social isolation, violence or abuse, housing insecurity, and exercise level of effort	Primary care clinics	Mixed methods	Cross-sectional
Sitapati et al [69], 2020	California, United States	Demonstrate supporting reliable care by standardized collection of SDOH data in EHR	Race, ethnicity, language, sexual orientation, and gender identity	Public hospitals	Quantitative	Prospective cohort
Stark et al [70], 2024	Texas, United States	Assess implementation of new EHR-based SDOH screening and resource referral	Health literacy, transportation issues, food insecurity, housing stability, financial strain, and legal concerns	Pediatric primary care clinic	Quantitative	Experimental (nonrandomized trial)
Wallace [71], 2020	Utah, United States	Evaluate a process for systematically identifying social needs during routine health service delivery	Housing insecurity, food assistance, transportation, mental health and addiction, employment, education, domestic violence, and abuse	ED	Mixed methods	Prospective cohort
Wang et al [72], 2021	California, United States	Develop measures to capture the use of SDOH screening	Transportation, financial strain, unstable housing, and food insecurity	Hospitals and ambulatory clinics	Quantitative	Retrospective cohort

^a^SDOH: social determinants of health.

^b^HRSN: health-related social needs.

^c^N/A: not available.

^d^SBIR: screening, brief intervention, and referral.

^e^ED: emergency department.

^f^EHR: electronic health record.

#### Settings and Study Population

There were 26 multisite studies and 17 single-site studies, while only 9/26 (35%) specified the number of sites. Major settings included hospitals (8/43, 19%), community health centers (8/43, 19%), primary care facilities (7/43, 16%), ambulatory clinics (4/43, 9%), and pediatric primary care facilities (4/43, 9%).

Studies focused either on adults (29/43, 67%), children (7/43, 16%), or all age groups (5/43, 12%). In addition, 2 articles did not mention the target age group. Data was collected from patients (35/43, 81%), health care providers (4/43, 9%), or both patients and providers (4/43, 9%). Studies (39/43, 91%) that had patient participants had a mean sample size of 35,314 (range 20-542,953). Studies (n=8) that had health care provider participants had a mean sample size of 52 (range 18-209).

#### SDOH Domains and Behavioral Risk Factors Covered

Domains covered by the studies included SDOH (37/43, 86%), behavioral risk factors (5/43, 12%), or both (1/43, 2%). A total of 36 unique SDOH factors were screened for, with most studies screening for multiple factors. Among the SDOH domains, housing (25/43, 58%), food (25/43, 58%), transportation (20/43, 47%), financial strain (13/43, 30%), and education (11/43, 26%) were the most common SDOH factors of interest. Among the behavioral risk factors, physical activity (10/43, 23%), alcohol (7/43, 16%), tobacco (5/43, 12%), and substance or drug abuse (3/43, 7%) were the most common factors of interest.

Many studies screened for multiple SDOH and behavioral risk factors (17/43, 40%), with a few focusing on 1 domain or factor (8/43, 19%). Other studies (18/43, 42%) did not indicate which SDOH domains or behavioral domains. The most screened SDOH domains were food insecurity (15/43, 35%), housing stability or insecurity (12/43, 23%), and transportation (12/43, 23%). The most screened risk factors were physical inactivity (6/43, 14%), tobacco use (4/43, 9%), alcohol (3/43, 7%), and drug use (1/43, 2%). Studies used a single (10/43, 23%) or a combination of validated screening tools (11/43, 26%), while others did not specify the screening tool (22/43, 51%).

### Strategies and Processes for Integrating Screening Tools

#### Overview

Among reported strategies for implementation of screening and referral tools (26/43, 61%) were providing support for staff (9/26, 35%). These supports included additions of documentation tools for tracking screening and referrals, and streamlining health care provider access to SDOH information.

Another strategy related to optimizing clinical workflows (17/26, 65%), with training at implementation start and technical assistance (12/17, 71%) being most common, while continued support (6/12, 50%) was also offered. Other strategies were the addition of patient navigators, community resource specialists, information specialists, social workers, or medical students to support workflows (8/17, 47%). Identifying a champion was another implementation strategy (5/17, 29%). A lesser-used strategy was collaboration with community-based organizations to provide immediate support (3/17, 18%). Studies frequently leveraged multiple implementation strategies.

#### Screening and Referral Processes

Table 2 provides a comprehensive snapshot of screening and referral characteristics, processes, and tools, and Epic modules used for developing integration tools in the EHR. The included studies mentioned implementing screening (16/43, 37%), referral (3/43, 7%), or both (24/43, 56%) for SDOH and modifiable risk factors.

Of the 3 reporting only referral rates, screening methods were not detailed. For example, Khanna et al [56] developed a clinical decision support tool that created automated e-referrals within the EHR. Similarly, a study done by Friedman et al [42] described how SDOH was identified and addressed by patient navigators. While navigators screened patients, rates were not reported. Hsu et al [51] reported that patients were screened and referred to a community resource specialist; however, screening rates were not reported.

Regarding the processes for screening, it was completed either before patient appointments (13/40, 33%) or before and during appointments (3/40, 8%). Modalities included patient self-screening before or during appointments (10/40, 25%) or by multiple providers (medical assistants, registered nurses, licensed practical nurses, nurse practitioners, social workers, unit clerks, physicians, and medical students; 9/40, 23%). Studies that reported the use of one provider role used medical assistants (4/40, 10%), nurses (4/40, 10%), or physicians (3/40, 7.5%). Of those that implemented screening (40/43, 93%), digital methods using the EHR (18/40, 45%) were the most common, while some studies used paper tools, which were later entered on the chart (5/40, 13%), or both digital and paper-based methods (5/40, 13%). Among studies that involved referrals after screening (27/43, 63%), resource lists and direct referrals to available programs or providers were either provided by providers (12/27, 44%), automated EHR referrals (8/27, 30%), or on patient request (2/27, 7%).

Tools reported for screening included the Protocol for Responding to & Assessing Patients’ Assets, Risks, and Experiences (PRAPARE; 6/43, 14%), Health Leads Screening Toolkit and Hunger Vital Sign Questionnaire (5/43, 12%), Accountable Health Communities’ health-related social needs Screening Tool (4/43, 9%), Alcohol Use Disorder Identification Test and National Academy of Medicine (3/43, 7% each) and other tools (1/43, 2%), or were not specified (22/43, 51%).

The most common mode of post-screening referral was staff or physician-initiated (12/43, 28%). Referrals included EHR automated (8/43, 19%), patient self-initiated (2/43, 5%), or not specified (21/43, 49%). About a third of the studies reported postreferral follow-up being completed (12/43, 28%).

**Table 2:** Details of screening and referral processes, including screening format, clinical staff involved, timing tools, postscreening referral and follow-up, and Epic modules used to integrate screening and referral (N=43).

Reported screening and referral modalities, tools, and integrations			Studies, n (%)		References
**Screening and referral characteristics**					
	Both screening and referral	24 (56)		[8,9,33-35,37,40,41,43-46,48-50,52-54,58,66-68,70,71]	
	Screening only	16 (37)		[32,36,38,39,47,55,57,59-65,69,72]	
	Referral only	3 (7)		[42,51,56]	
**Screening administration**					
	Patient	9 (21)		[9,40,43,46,53,59,62,63,69]	
	Multiple staff (medical assistants, registered nurses, licensed practical nurses, nurse practitioners, social workers, unit clerks, physicians, and medical students)	8 (19)		[32,37,44,50,58,67,68,71]	
	Medical assistant	3 (7)		[34,35,38]	
	Staff	3 (7)		[54,65,70]	
	Nurses	2 (5)		[33,45]	
	Patient navigator	2 (5)		[42,66]	
	Clinician	1 (2)		[72]	
	Physician	1 (2)		[52]	
	Not specified	14 (33)		[8,36,39,41,47-49, 51,55-57,60,61,64]	
**Screening format**					
	Digital (completed on computer, phone, or tablet)	18 (42)		[32,33,37,38,45,46,48,50,52,58,62,63,65,66,68-71]	
	Paper	5 (12)		[9,34,40,41,43]	
	Both digital and paper	5 (12)		[35,44,53,60,67]	
	Not specified	15 (35)		[8,36,39,42,47,49, 51,54-57,59,61,64,72]	
**Screening timing**					
	Before appointment	13 (30)		[9,32-34,37,40,41,43,44,53,60,62,65]	
	Before or during the appointment	4 (9)		[45,50,70,71]	
	Not specified	26 (61)		[8,35,36,38,39,42,46-49,51, 52,54-59,61,63,64,66-69,72]	
**Tools for screening^a^**					
	Protocol for Responding to & Assessing Patients Assets, Risks, and Experiences (PRAPARE) tool	6 (14)		[32,38,44,47,49,68]	
	Health Leads Screening Toolkit	5 (12)		[40,41,49,68,71]	
	Hunger Vital Sign Questionnaire	5 (12)		[9,35,49,58,68]	
	Accountable Health Communities HRSN^b^ Screening Tool	4 (9)		[32,46,49,68]	
	Alcohol Use Disorder Identification Test (AUDIT)	3 (7)		[33,37,63]	
	National Academy of Medicine	3 (7)		[38,44,47]	
	Montefiore’s 10 Question SDOH^c^ Survey	2 (5)		[49,68]	
	Single Item Literacy Screener	2 (5)		[49,68]	
	UCLA^d^ Three Item Loneliness Scale	2 (5)		[49,68]	
	WE CARE screening instrument	2 (5)		[9,43]	
	American Academy of Family Physicians Screening Tool	1 (2)		[35]	
	CF Social Needs Screening Tool	1 (2)		[53]	
	Drug Abuse Screening Test (DAST-10)	1 (2)		[63]	
	Epic Social Factors Screen	1 (2)		[35]	
	Epic Tobacco Use Assessment	1 (2)		[54]	
	Exercise Vital Sign	1 (2)		[68]	
	HealthBegins Upstream Risk Screening Tool	1 (2)		[68]	
	Housing Stability Vital Sign	1 (2)		[68]	
	Humiliation, Afraid, Rape, Kick (HARK) Screening Tool	1 (2)		[68]	
	National Institute on Alcohol Abuse and Alcoholism	1 (2)		[33]	
	Patient Health Questionnaire	1 (2)		[37]	
	Prescription Misuse Question	1 (2)		[37]	
	Physical Activity Vital Sign (PVAS)	1 (2)		[62]	
	USDA’s^e^ Household Food Security Survey	1 (2)		[45]	
	Not specified	22 (51)		[8,34,36,39,42,48,50-52, 55-57,59-61,64-67,69,70,72]	
**Mode of post-screening referral provided**					
	Staff- or physician-initiated	12/ (28)		[33,35,40,42,43,45,46,51,53,66,67,70]	
	Electronic health record (EHR) automated	8 (19)		[41,48,49,54,56,58,68,71]	
	Patient self-initiated	2 (5)		[9,38]	
	Not specified	21 (49)		[8,32,34,36,37,39,44,47,50,52,55,57,59-65,69,72]	
**Follow-up to referral services completed**					
	Follow-up completed	12 (28)		[9,40-43,50-53,66,70,71]	
	Not specified	31 (72)		[8,32-39,44-49,54-65,67-69,72]	
**Epic modules used for screening and referral tools^a^**					
	Best Practice Alerts (BPAs)	7 (16)		[33,37,54,63,65,67,70]	
	MyChart	6 (14)		[32,53,64,65,67,70]	
	Epic SmartPhrases	4 (9)		[33,43,54,58]	
	SDOH Wheel	3 (7)		[50,58,60]	
	NowPow	2 (5)		[49,68]	
	Epic SDOH module	2 (5)		[34,59]	
	Not specified	26 (60)		[8,9,35,36,38-42,44-48,51, 52,55-57,61,62,66,68,69,71,72]	

^a^Due to multiple counts, the total may not add up to n=43 studies.

^b^HSRN: health-related social needs.

^c^SDOH: social determinants of health.

^d^UCLA: University of California, Los Angeles.

^e^USDA: United States Department of Agriculture.

### Epic Modules Used for Integrating Screening Tools

Table 2 shows that less than half of the studies mentioned the use of Epic modules (17/43, 40%) within the intervention. The most common modules included BPAs (7/43, 16%), MyChart (6/43, 14%), SmartPhrases (4/43, 9%), the SDOH wheel (3/43, 7%), Epic SDOH module (2/43, 5%), and NowPow (2/43, 5%).

### Outcomes Reported From EHR Integrations

#### Overview

This section presents the outcomes of the varied integrations, key findings, and the domains of SDOH and modifiable risk factors as part of the EHR integrations. Pooled results and individual study results are presented in Table 3 and Multimedia Appendix 5.

**Table 3:** Mean screening and referral rates across interventions.

Pooled rates across screening and referral modalities		Mean% (SD)	Range, %	References
Pooled screening rates reported across all studies		50 (33)	0.03-93	—^a^
**Screening: mode of administration**				
	Self-administered	62 (31)	16-95	[9,32,40,43,46,53,59,62,63,69]
	Nurse administered	58 (19)	30-74	[32,33,45,58]
	Medical assistant administered	44 (36)	2-83	[34,35,38,58]
	Physician administered	58 (19)	0.09-91	[52,58,72]
	Other staff administered	50 (28)	18-71	[54,70,71]
	Patient navigator administered	17	17	[66]
	Multiple clinical staff administered^b^	52 (36)	2.5-97	[37,44,50,65,67,68]
Pooled referral rates across all studies		36 (25)	6-79	—
**Referral: mode of administration**				
	Automated referral initiated by the EHR^c^ in response to certain criteria	32 (18)	18-71	[41,48,49,54,56,58,68,71]
	Patient-initiated referral	14 (11)	6-22	[9,38]
	Provider-initiated referral	45 (26)	6-79	[33,35,40,42,43,45,46,51,53,66,67,70]
**Referral type**				
	Referral to paper or web-based resources	28 (27)	6-79	[9,38,42,43,45,46,49,53,54,58,67,68]
	Referral to another provider or program	45 (19)	18-71	[33,35,40-42,48,51,54,56,66,70,71]
	Referral refused by patient	22 (4)	18-26	[38,56,70]

^a^Not available.

^b^Multiple clinical staff include medical assistants, registered nurses, licensed practical nurses, nurse practitioners, social workers, unit clerks, physicians, medical students, and unspecified staff.

^c^EHR: electronic health record.

#### Screening and Referral Rates: Pooled Estimates

We provide pooled high-level estimates of outcomes of EHR integration of screening and referral tools. Given variations across settings, domains, interventions, and populations, the range and mean (SD) of outcomes are provided. Table 3 presents the screening rates, referral rates, referral to paper or online resources, referral to another provider, and refused referral rates across all studies.

The mean physician-administered screening rate was 58% (SD 19%, range 1%-91%) for 3 studies, while patient self-administered screening across 10 studies was 62% (SD 31%, range 16%-95%). Among the 4 studies that used nurse-facilitated screening, the mean rate was 58% (SD 19%, range 30%-74%). Across the 4 studies where screening was administered by a medical assistant, the pooled mean rate was 44% (SD 36%, range 2%-83%; see Table 3).

Across the 17 studies reporting patients at-risk for at least one SDOH domain, the pooled mean was 37% (SD 29%, <1%-98%). Most studies also reported SDOH and behavioral risk factor needs that were most frequently identified by patients during the intervention period. A total of 6 studies reported a change in screening rates using pre- and postimplementation outcomes. Among these, the pooled mean screening rate increase was 47% (SD 30%, 6%-80%). In addition, 2 studies reported changes in referral rates after the intervention, noting an increase of 6% and 20%, respectively.

Among the individual studies, reported outcomes highlight improvements in screening rates across different settings, both in documentation and addressing SDOH and modifiable risk factors (see Multimedia Appendix 4). Significant increase in screening rates was observed with patient-filled previsit screening using MyChart. MyChart boosted ambulatory screening rates from 0.4% to 15.9% and inpatient rates from 0% to 66% in 1 study [32], while in another 3, at least 70% to 90% patients completed screening before or during the appointment [53,65,70]. In another study, screening rates rose from 15% to 62%, with discussions of results increasing from 61% to 81%, with fulfilled referrals from 37% to 57% [35]. Conversely, a documentation study reported that rare recording of social history, with free-text fields having higher use [72].

#### Referrals for SDOH and Modifiable Risk Factors Identified During Screening

In comparison to screening, referral rates from applicable studies were lower, with a pooled mean rate across all the studies (n=43) being 36% (SD 25%, 6%-79%; see Table 4). Referral rates to resources, programs, or providers also varied by referral type. Among 12 studies where referrals were provided by staff or physicians, the pooled mean referral rate was 45% (SD 26%, 6%-79%). In the 8 studies that opted for EHR automated referrals, the pooled mean rate was 32% (SD 18%, 18%-71%). Pooled mean referral rate for 12 studies with patients who received a paper or web-based resource list was 28% (SD 27%, 6%-79%), while for the 10 studies that referred patients to a provider or program, it was 45% (SD 19, 18%-71%). Pooled refusal rate for the 3 studies for patient declined referrals was 21% (SD 4%, 18%-26%).

Among individual studies, there was a wide variation in referrals. In a study where almost all patients had one or more SDOH needs, 19% were referred [44], while in another, 25% of patients identified with SDOH needs received a referral [49] (see Multimedia Appendix 4). In another study, patient navigators made 18,284 unique community resource referrals for 66% of the 11,273 patients with SDOH needs [42]. For behavioral risk factors, a study showed that 71% of patients had a tobacco treatment appointment scheduled [54].

**Table 4:** Lessons learned from integrating social determinants of health (SDOH) and risk factor domains in the Epic electronic health record (EHR).

Themes and subthemes			Related lessons
**Theme 1: Tailored processes for effective screening and referral**			
	Embedding SDOH tools in EHRs is not sufficient for full adoption of screening and referral [38,43,59,72].	Dedicated SDOH fields and tools in the EHR were insufficient to drive increased screening. Effective communication and multiple delivery methods are valuable to promote uptake.	
	Screening is feasible with the right workflows and support, but challenging to adopt [8,9,40,43,44,49-52,55,60-62,65,66,70,71].	A clear purpose must be emphasized to integrate screening. Staff recognize the value of SDOH screening but find it challenging to integrate it into their workflows. Efficient workflows and strong leadership support are required to build screening into practice. Screening burden can lead to provider fatigue if too time-consuming and frequent. Implementing and maintaining SDOH screening was challenging. Under-resourced clinics may struggle to sustain processes over longer periods of time, even if valuable to patients. Importance of identifying and addressing organizational and administrative barriers before implementation. Designated staff (social workers, exercise or behavior change experts) are needed to coordinate screening and referral efforts in the clinic.	
	Context matters: implementation approaches must be tailored to the contextual realities at each location [35,38,43,47,56,58,59,61-64].	Different screening and referral approaches may be suitable. Method: paper-based, electronic, or verbal. Timing: before or during appointment. Provider: doctor, nurse, social worker, assistant, or resident.	
		Screening and referral rates varied widely across locations, providers, methods, and populations. More likely in-person, documented by a medical assistant. Less likely if confidence in and availability of trusted resources to refer to are limited. The majority of screening was completed by certain providers or clinics, while others had much lower rates. Non-native speakers were less likely to be screened or report needs.	
	Iterative adaptation can improve implementation [9,32,45,47,52,53].	Rapid testing and refinement of processes enhanced implementation Screening rates increased significantly after the plan, do, study, and act (PDSA) cycles.	
**Theme 2: Implementing SDOH and modifiable risk factor screening and referral positively impacts care**			
	SDOH screening and referral improve the quality and equity of health care, providing more holistic care [40,42,43,48,49,51,54,62,69].	Addresses issues not identified in routine care that can impact health outcomes. Financial care models that benefit from preventive strategies are key to the sustainability of SDOH screening and referral.	
	Screening and referral helped simplify and increase connection to resources [34,39,40,46,59].	Implementation led to examination of clinic processes regarding social needs, financial assistance, and social worker consultation. Identification of needs and provisions does not guarantee connection to resources or mitigation of needs. Community health workers played a crucial role in connecting patients with appropriate resources. List of trusted resources and professionals to refer to increases confidence and ability to address SDOH needs.	

#### Major SDOH and Behavioral Domains, and Patient Acceptability

The most common SDOH challenges identified by the patients included food insecurity (15/43, 35%), housing instability (9/43, 21%), and medication affordability (6/43, 14%). The most common reported at-risk behavioral factors were physical inactivity (3/43, 7%), alcohol (3/43, 7%), and tobacco use (3/43, 7%; see Table 4). Among significant findings from individual studies, mental health conditions, including stress. was the most identified SDOH domain, affecting a third of all patients [34], while depression screening accounted for almost 60% of positive screens [37]. Related to mental health, one large study with more than 100,000 patients showed that while less than 1% of the patients were identified with SDOH needs, psychosocial circumstances accounted for 73% of the codes [55] (see Multimedia Appendix 4).

One study reported the integration of a homelessness screen under the California State Bill 1152 (SB1152), which resulted in identifying double the number of patients in a large hospital emergency department over a year serving 75,000 patients annually [39]. In addition, patients with unique visits rose by 143%, discharges increased by 8%, and admissions decreased by half, from 18% to 9%. The intervention was also effective in attracting a 12% cumulative increase in visits by the Black, Asian, and Hispanic races, while patients with only 1 emergency department visit decreased by 6%, while those with 4 or more visits increased by 10% [39].

#### Patient and Provider Experiences

Qualitative findings added insights into the experiences and perceptions of both patients and health care providers regarding SDOH screening. One study identified 3 key facilitators for implementing systematic SDOH screening: external motivators, internal advocates, and flexible approaches to developing screening workflows [47]. Another study found that patients generally had a neutral or positive perspective on SDOH screening, believing that providers should be aware of their social situations [50].

Provider perspectives also highlighted the challenges and benefits of SDOH screening. One study reported 72% of health care professionals being somewhat familiar with SDOH screening, and 93% supported its incorporation into standard care [57]. However, providers noted that screening for health behaviors was part of routine care, than for SDOH. Another study found that 90% of physicians believed SDOH information could improve patient care and therapeutic relationships, but only a third felt confident that having SDOH information would influence medical decision-making [34].

### Key Barriers and Facilitators

#### Organizational Facilitators

Studies described a broad range of facilitators that helped with implementing Epic-based SDOH screening and referral tools in the EHR. Most facilitators were either related to the organizations and their staff or to the systems and processes used for screening and referral. Leadership support and staff buy-in for SDOH screening [8,9,32-34,38,41,53,56,63-65,69], team champions and alternative care providers (eg, social workers, physical activity specialists) directly on-site, were key to increasing screening and referral rates [8,9,34,38,40,41,44,47,48,51,53,56,60,63-65,68].

#### Process and Workflow Facilitators

Workflow flexibility context-based adaptations were [8,9,35,40,46-48,52,53,58,63], education and training on the value and process of SDOH screening [8,40,45,56,60,69,70,72], well-defined roles and protocols [34,38,40,51,57,60,63,70] were key workflow-related facilitators. Integrations were also enhanced by leveraging preexisting clinic workflows and standardized EHR tools [9,32,40,46,49,54,58,60,64,66,69,70,72], involving multiple providers (resident physicians, physician assistants, and nurses) [37,39,50,52,54,58,59], and with automated reminders [32,70].

#### Barriers Related to Processes, Resource Constraints, and Individual Concerns

There were 3 major categories of implementation barriers noted: unclear processes, lack of resources, and individual concerns. A major challenge was the lack of consensus for how to capture SDOH needs in the Epic EHR, including overly complex screening tools and the lack of standardized questionnaires [35,38,43,51,57,60,68,70,72]. Furthermore, providers were either reluctant to complete SDOH screening without a clear plan for addressing identified concerns [34,38,48,51,52,60,66] or their knowledge and confidence in referring patients to resources were limited [9,34,45,48,57,60,63,68]. These barriers were further compounded by difficulties in monitoring referral progress [32,42,46].

Resource barriers centered around limited time, competing priorities, inefficient workflows, devices such as tablets, and support staff to complete SDOH screening and referral [8,9,33,34,44,45,47,48,55,57,63,64,66,68,69]. High staff turnover also contributed to high resource demands from increased training burden [9,40,43,70]. Individual barriers included data privacy concerns and fear of negative impact [34,44,55,59,60,68,71], poor literacy of digital health tools [9,69], and screening tools being only available in English [9,46,49,70].

## Discussion

### Overview and Value-Add

This review synthesizes evidence on methods and processes for integrating SDOH and modifiable risk factors into the care processes, along with contextual evidence on tools, documentation, and workflows in the largest and most globally adopted EHR. By understanding the patient's social context and modifiable risk factors, care plans can be directed towards root causes, helping achieve the grander aim of patient-centered, equitable health systems.

This study provides a granular synthesis of evidence on efforts related to SDOH integrations in a widely adopted and capable EHR [11]. Overall, the review shows significant improvements in screening rates and identification of social needs in clinical settings, while highlighting perceived benefits and challenges from patient, provider, and health system perspectives, which helps build a deeper understanding of the effectiveness and improvements needed in incorporating the SDOH approach in routine clinical practice.

We address a gap in the literature for researchers and practitioners for optimizing and informing future applications in Epic and other EHRs. Lessons and recommendations from the review are generalizable to other EHRs and contexts, as the challenge and aim of targeting equity through integrating SDOH domains in eHealth systems is a shared global goal. We discuss and reflect on complexities, adaptations, and challenges here, while contrasting with findings from related recent systematic reviews.

### Summary of Evidence

#### Trends and Implications From the Current Evidence

We observed a recent upward trend in research on integrating tools that address both SDOH and modifiable risk factors, aligning with health systems’ recent focus toward integration of both patient and community-level SDOH data into EHRs to support equity-oriented health planning [73]. We could not find when exactly Epic included SDOH domains within its tools; however, their recent documentation on the use of the SDOH wheel, tools, customization, and modification of the domains refers to the 2015 National Academy of Medicine’s guideline on documenting SDOH factors in EHRs [74,75]. Despite Epic being among the most globally adopted EHRs, we could not identify peer-reviewed or gray literature outside of the United States, which limits the generalizability of the findings, while underscoring the need for international research in this area. However, given that Epic’s SDOH tools are global, customizable, and, along with the need for contextual integrations, this should not be viewed as a serious limitation of the presented findings.

The predominance of retrospective cohort studies highlights the usability of EHRs for evaluation. However, experimental designs would add robust evidence of effectiveness in real-world settings. In addition, the relatively low proportion of qualitative research points to the lack of contextual insight from such integrations. Furthermore, studies were mainly from large, multisite hospital systems, suggesting that perhaps SDOH integrations are feasible in better-resourced settings. While this is comprehensible, the argument for equity remains unaddressed for populations that need and would benefit the most from such integrations.

#### Lessons Learned for Improving the Impact of Integration Strategies

Screening for SDOH domains and modifiable risk factors positively impacted the quality and equity of care. Such initiatives helped promote connections with resources, whether internal or external to the health system. Proximal to such connections was the examination of clinical processes that could screen for social needs and connect to consultations with allied social workers. In this regard, community health workers’ role was important in connecting patients to appropriate resources, which resulted in enhancing patient trust and confidence towards addressing their needs. Using qualitative content analysis, we categorized the key takeaways into themes and subthemes (see Table 4).

Among key enablers to overcome expected barriers in integration of SDOH and related tools in EHRs, organizational support, particularly leadership engagement and staff buy-in, is foundational. Dedicated champions and on-site alternative care providers, such as social workers, further enhance uptake of integrated tools. Flexibility to adapt workflows to the local context emerged as a critical factor. Process-level enablers included role clarity, standardized protocols, and targeted staff training. Leveraging existing clinic workflows and Epic functionalities can also be used to streamline integrations.

Towards integration of SDOH domains in the EHR, 2 interrelated strategies included workflow optimization and enhanced support. Workflow improvements focused on staff training, introducing new roles such as care navigators and social workers, and engaging clinical champions to drive adoption. In parallel, support enhancements involved customizing EHR documentation, building referral linkages, and integrating tools. Within EHR enhancements, the prominence of and acceptability for self-administered screening through patient-facing technology such as MyChart is a major finding. While this can save provider time, digital literacy can be a limiting factor.

While staff and leadership buy-in, clear purpose and efficient workflows lay the foundation of integration initiatives, resources, including designated staff and targeting organizational barriers, were important facets. In addition, iterative adaptations were found to be effective in improving implementations over time, for example, with multiple PDSA (plan, do, study, and act) cycles.

One resounding message was that a one-size-fits-all approach may not be effective, particularly for motivating providers to chart requisite information. This is expected as changes in EHRs can place an added burden on providers’ time [76]. Ensuring and capturing quality data, patient and provider experiences, and clinical local area are of significant importance. Furthermore, while tailoring and customization can be at different levels and workflows, flexibility is needed within built-in and customized tools with continued technology and evaluation support [14]. Specific contextual needs of the clinical setting, including characteristics of the patient population and resources, may necessitate developing unique workflows. For example, while a proportion of studies used vendor-supplied tools in the EHR, others developed their own tools tailored to their settings.

### Recommendations for Future Research

#### Need for Standardizing SDOH and Modifiable Risk Factors Domains

While the Epic SDOH wheel provides an opportunity to document SDOH and modifiable risk factors, capture SDOH domains, the module has been primarily developed for the health systems in the target US health system. In our work at the AHS Provincial Population and Public Health Connect Care Working Group, we were not able to use the SDOH wheel as an off-the-shelf tool, with several screening domains needing adaptation to meet the Alberta context.

Standardized, reliable data are critical toward understanding upstream social determinants in any population to shape evidence-driven policy and enhance impact [13-15,17]. Gaps within and related to standardization, such as SDOH domains, validated questions, brevity, actionability, cultural appropriateness, workflow integration, and community linkages, have been pointed out by multiple reviews [13,14,17]. Ganatra et al [15] propose a comprehensive tool to provide actionable data through standardized data collection, enabling risk stratification and designing targeted initiatives to address health inequities at both individual and population levels.

Consensus building on standardized tools for SDOH domains and modifiable risk factors, in their particular context, is an important consideration [14,15,77]. In Canada, the SPARK (Screening for Poverty and Related Social Determinants and Intervening to Improve Knowledge of and Links to Resources) tool [78] is a validated, evidence-based questionnaire to help primary care and community-based organizations consistently collect and act on patients’ social needs.

#### Workflows, Data Usage, and Impact Evaluation

Related to data collection, extraction and linkage, research should explore patient and provider perspectives on collecting and integrating data on SDOH and modifiable risk factors. Methods for capturing and analyzing EHR data, including machine learning approaches are important areas, which require development, validation and reproducibility [15,38]. Further linking disparate datasets, such as EHR, administrative and community data sources is another method for creating multidimensional patient profiles for clinical decision support [13,14,77].

Further research is also needed for developing evidence-based pathways for integrating SDOH data into real-world patient care workflows, improving risk prediction algorithms, treatment plans, targeted referrals, and utilization of SDOH data to inform clinical decision-making to address health-related social needs, and reducing disparities [15,38,77,79]. In addition, mixed methods studies can add to the context of implementing tools that are feasible and carry long-term impact. Implementation science studies, usability testing, and human-computer interaction studies can help arrive at pragmatic solutions to capture risk data in EHRs.

Research is also needed to capture medium and longer-term health and economic benefits of screening and referrals for SDOH screening in clinical settings [17,80]. Last but not least is incorporating the quintuple aim of enhancing patient experience, improving population health, reducing costs, targeting health equity, and provider well-being in all such EHR integrations [81].

### Limitations

This scoping review attempted to synthesize the breadth of knowledge related to Epic as one of the largest, globally adopted EHRs, keeping SDOH and modifiable risk factors as important upstream factors affecting health. One of the major limitations in this work is arguably generalizability. First, findings of the review from the Epic EHR may not be generalizable to other EHRs such as Cerner. However, we contend that integration issues within other EHR systems, built-in and modifiable modules, and, more importantly, implementation and change management challenges are of a similar nature. Generalizability may also be limited due to the wide variations in the settings, approaches, tools, and evaluation methods that different studies used. This is expected in scoping reviews, as the main aim of such synthesis is to cast a wide net to capture the breadth of literature.

When it comes to capturing domains within the SDOH approach, there is an inherent assumption that these are to be captured and addressed in primary care. However, there is a recent shift toward addressing these upstream factors across the health system. For example, the World Health Organization’s Healthy Hospitals approach aims at promoting the health of patients, staff, and the community that it serves [82]. Hence, while these findings are limited to larger EHRs, they are relevant for researchers and practitioners offering care at all levels.

Our objective was to synthesize global literature on the subject; however, we could not find published peer-reviewed or gray literature articles and reports outside of the United States. However, learnings can be of benefit to international communities of research and practice using the Epic EHR. Given the importance of the wider SDOH, this is a major gap that researchers can target in the future. Since many studies did not report on the entire pathway for screening and referral, we could not separate out lessons for each step. For example, while screening may work well in a setting, connections to and updates from external services surfaced as a known challenge. In addition, many studies do not report on the specific Epic EHR modules and tools, which limited our syntheses.

While we found aggregating effectiveness rates from studies as feasible and valuable [30], pooled outcomes in this review should be interpreted with caution due to variations in methods for calculating screening and referral rates among the studies (eg, percentage of all patients, percentage of target population only, or any other), the duration of data collection, and availability of pre-implementation baseline rates, and most importantly the context of the EHR integration itself. To the latter, context can relate to settings, the level of care, specialty, staff involved, and resources available for complex implementation challenges and change management. While we attempted to derive pooled estimates, heterogeneity within reported statistics is expected in any exercise involving meta-analyses [30]. This could be due to methodological, clinical, or statistical factors.

Other important limitations related to our methods were the search, which included English language articles only, and the lack of critical appraisal of the included studies. We did not include a formal critical appraisal of the methodological quality of included studies, as our main objective was to map the range and characteristics of Epic-integrated tools. This limits conclusions about the strength of evidence underlying specific tools or implementations.

This review did not focus on the tools and constructs for SDOH and modifiable risk factors, while capturing and presenting variations in the literature. This is an important policy and research area, requiring consensus on priorities of the health system, but also of the society at large.

### Conclusions

Upstream SDOH and modifiable risk factors are being increasingly targeted for improving population health outcomes and the provision of equitable care. In this review focused on the Epic EHR, we found a sharp upward trend in the uptake, optimization, and integration of tools to screen, offer care, and referral for appropriate services within or outside the health care system. The Epic EHR incorporated the “SDOH wheel” for capturing such domains; however, customized integrations beyond built-in tools seem to be the norm. We captured a variety of built-in tools, customizations, and optimizations in this review. We also felt that there is an increasing need for standardization of the SDOH domains within and across EHRs. Future research is crucial for context-based standardization of tools, best practices, data on outcomes and impact for learning, and developing targeted interventions.

## Appendix

Multimedia Appendix 1 Search planning document for all data sources.

Multimedia Appendix 2 Preferred Reporting Items for Systematic Reviews and Meta-Analyses (PRISMA) checklist.

Multimedia Appendix 3 Search strategy.

Multimedia Appendix 4 Specific interventions, EPIC tools and reported results.

Multimedia Appendix 5 Comprehensive study findings.

## Abbreviations

AHS: Alberta Health Services
EHR: electronic health record
PDSA: plan, do, study, and act
PICOS: population, intervention, comparison, outcome, and study type
PRAPARE: Protocol for Responding to & Assessing Patients’ Assets, Risks, and Experiences tool
PRISMA-ScR: Preferred Reporting Items for Systematic Reviews and Meta-Analyses extension for Scoping Reviews
SBIR: Screening, Brief Intervention and Referral
SDOH: social determinants of health
SPARK: Screening for Poverty and Related Social Determinants and Intervening to Improve Knowledge of and Links to Resources
