# Supplementary material for: Integration of Screening and Referral Tools for Social Determinants of Health and Modifiable Lifestyle Factors in the Epic Electronic Health Record System: Scoping Review
Source: J Med Internet Res. 2025 Sep 15;27:e73615. doi: 10.2196/73615 (PMC12494108; doi:10.2196/73615)
Supplement: Multimedia Appendix 1 [file jmir_v27i1e73615_app1.docx]

# Integration of Social Determinants of Health and Lifestyle Factors as Screening Tools in Epic EHR

## Search Planning Document

### Search Terms

| **Concept** | **Synonym** |
| --- | --- |
| Social Determinants of Health ^1^ | “social determinants of health” [Keyword, MeSH]; SDOH [Keyword]; “health social determinant*” [Keyword]; “health structural determinant*” [Keyword]; “structural determinants of health” [Keyword]; |
| Behavioural & Lifestyle Factors | “behavioral factor*” [Keyword]; “behavioural factor*” [Keyword]; “life style” [Keyword, MeSH]; “lifestyle factor*” [Keyword]; “behavior support” [Keyword]; “behaviour support” [Keyword]; “health behavior*” [Keyword]; health behavior [MeSH]; “health behaviour*” [Keyword]; “social risk factor*” [Keyword]; “social factor*” [Keyword]; social factors [MeSH]; “behavioral health risk factor*” [Keyword]; “behavioural health risk factor*” [Keyword]; “behavioral health” [Keyword]; “behavioural health” [Keyword]; |
| Screening, Brief Intervention and Referral | “screening, brief intervention and referral” [Keyword], SBIR [Keyword]; SBIRT [Keyword]; SBI [Keyword]; “screening brief intervention referral to treatment” [Keyword]; |
| Economic Stability ^5^ | “economic stability” [Keyword, MeSH]; “economic instability” [Keyword]; income [Keyword, MeSH]; “income equality” [Keyword]; “income inequality” [Keyword]; “socioeconomic status” [Keyword]; SES [Keyword]; social class [MeSH]; “socioeconomic factor*” [Keyword]; socioeconomic factors [MeSH]; poverty [Keyword, MeSH]; “financial resource strain” [Keyword]; employment [Keyword, MeSH]; unemployment [Keyword, MeSH]; “basic needs” [Keyword] |
| Neighborhood & Built Environment ^3 4 6^ | Neighborhood* [Keyword]; neighbourhood* [Keyword]; communit* [Keyword]; residence characteristics [MeSH]; “built environment” [Keyword, MeSH]; “food insecurit*” [Keyword]; “food rationing” [Keyword]; food insecurity [MeSH]; transportation [MeSH]; “transportation needs” [Keyword]; commuting [Keyword]; “living arrangement*” [Keyword]; “living situation” [Keyword]; “food access” [Keyword]; environment [Keyword, MeSH]; “environmental condition*” [Keyword]; “housing stability” [Keyword]; |
| Health & Health Care ^7^ | “health insurance” [Keyword]; insurance, health [MeSH]; “insurance status” [Keyword]; stress [Keyword]; “psychological stress” [Keyword]; stress, psychological [MeSH]; depression [Keyword, MeSH]; “mental health” [Keyword, MeSH]; “mental illness” [Keyword]; “mental disorder*” [Keyword]; mental disorders [MeSH]; “emotional abuse” [Keyword, MeSH]; “psychosocial factor*” [Keyword]; “psychological comorbidit*” [Keyword]; “psychological abuse” [Keyword]; education [Keyword, MeSH]; “health literacy” [Keyword, MeSH]; |
| Social & Community Context ^8^ | “social context” [Keyword]; “social environment” [Keyword, MeSH]; “community context” [Keyword]; “social connection*” [Keyword]; “intimate partner violence” [Keyword, MeSH]; “intimate partner abuse” [Keyword] |
| Smoking/Tobacco Use | Smoking [Keyword, MeSH]; “tobacco use” [Keyword, MeSH]; “tobacco consumption” [Keyword]; |
| Alcohol Use | “alcohol use” [Keyword]; alcohol drinking [MeSH]; “alcohol intake” [Keyword]; |
| Physical Activity/Inactivity ^2^ | “physical activity” [Keyword]; exercise [Keyword, MeSH]; “physical inactivity” [Keyword]; inactiv* [Keyword]; “lack of physical activity” [Keyword]; “sedentary behavior” [Keyword, MeSH]; “sedentary behaviour” [Keyword]; “sedentary lifestyle” [Keyword]; “sedentary time” [Keyword]; sedentary [Keyword] |
| Electronic Health Records | “electronic health record*” [Keyword]; electronic health records [MeSH]; EHR [Keyword]; “electronic medical record*” [Keyword]; EMR [Keyword]; epic [Keyword]; “epic care” [Keyword]; “epic EHR” [Keyword]; “epic EMR” [Keyword]; “epic electronic health record*” [Keyword]; “epic electronic medical record*” [Keyword] |

^1^ Determinants of Health search filters, [Determinants of Health - Health Sciences Search Filters - Subject Guides at University of Alberta Libraries (ualberta.ca)](https://guides.library.ualberta.ca/search-filters/determinants-of-health), will also be utilized where applicable

^2^ [Exercise and Physical Activity - Google Docs](https://docs.google.com/document/d/1hxz5nEuW1Zvl-xQ_qwq8VqN0R817IOzfyRzSpNkfj9g/edit#heading=h.qi55eeyvgzy9) search filter will also be utilized where applicable

^3^ [Food Security - Google Docs](https://docs.google.com/document/d/1Kt-x9_4PJ3wynK6HgqAqJet8WBPLc1OrUl1jLevQQeA/edit) search filter will also be utilized where applicable

^4^ [Transportation Deficit - Google Docs](https://docs.google.com/document/d/1PoqBR0qumwMbevxEyUeQCve7bSEu1pegaKUjpPsJEEg/edit) search filter will also be utilized where applicable

^5^ [Poverty - Google Docs](https://docs.google.com/document/d/1CWzA20kP8arGnhVOh-LTN2aQji0wdXQ3vaRJc9PEf3c/edit) search filter will also be utilized where applicable

^6^ [Houseless People-Homelessness - Google Docs](https://docs.google.com/document/d/1m9whGdEeGulXhlK0qLLKOlbZfEbX6NpnfFs3OpG94p8/edit) search filter will also be utilized where applicable

^7^ [Educational Attainment - Google Docs](https://docs.google.com/document/d/1dRhVX_1XpOrXFhojatjyyFlvMyZFYKaWQW1ov_uIXUQ/edit) search filter will also be utilized where applicable

^8^ [Domestic Violence - Google Docs](https://docs.google.com/document/d/1dWDUkizzTf3xts2cc_Q3a9G_OE9g018UF1V55rr6TcI/edit) search filter will also be utilized where applicable

### Databases

MEDLINE (Ovid); EMBASE: PubMed; CINAHL

### Limits Applied

Age: all

Geography: open

Language: English

Publication Type: **exclude** commentaries, editorials, letters, conference abstracts

### Suggested Search Strings – Keywords (PubMed & CINAHL)

(“social determinants of health” OR SDOH OR “health social determinant*” OR “health structural determinant*” OR “structural determinants of health”) AND (smoking OR “tobacco use” OR “tobacco consumption”) AND (“electronic health record*” OR EHR OR “electronic medical record*” OR EMR OR epic OR “epic care” OR “epic EHR” OR “epic EMR” OR “epic electronic health record*” OR “epic electronic medical record*”)

(“social determinants of health” OR SDOH OR “health social determinant*” OR “health structural determinant*” OR “structural determinants of health”) AND (“alcohol use” OR “alcohol intake”) AND (“electronic health record*” OR EHR OR “electronic medical record*” OR EMR OR epic OR “epic care” OR “epic EHR” OR “epic EMR” OR “epic electronic health record*” OR “epic electronic medical record*”)

(“social determinants of health” OR SDOH OR “health social determinant*” OR “health structural determinant*” OR “structural determinants of health”) AND (“physical activity” OR exercise OR “physical inactivity” OR inactiv* OR “lack of physical activity” OR “sedentary behavior” OR “sedentary behaviour” OR “sedentary lifestyle” OR “sedentary time” OR sedentary) AND (“electronic health record*” OR EHR OR “electronic medical record*” OR EMR OR epic OR “epic care” OR “epic EHR” OR “epic EMR” OR “epic electronic health record*” OR “epic electronic medical record*”)

(“social determinants of health” OR SDOH OR “health social determinant*” OR “health structural determinant*” OR “structural determinants of health”) AND (“behavioral factor*” OR “behavioural factor*” OR “life style” OR “lifestyle factor*” OR “behavior support” OR “behaviour support” OR “health behavior*” OR “health behaviour*” OR “social risk factor*” OR “social factor*” OR “behavioral health risk factor*” OR “behavioural health risk factor*” OR “behavioral health” OR “behavioural health”) AND (“electronic health record*” OR EHR OR “electronic medical record*” OR EMR OR epic OR “epic care” OR “epic EHR” OR “epic EMR” OR “epic electronic health record*” OR “epic electronic medical record*”)

(“social determinants of health” OR SDOH OR “health social determinant*” OR “health structural determinant*” OR “structural determinants of health”) AND (“screening, brief intervention and referral” OR SBIR OR SBIRT OR SBI OR “screening brief intervention referral to treatment”) AND (“electronic health record*” OR EHR OR “electronic medical record*” OR EMR OR epic OR “epic care” OR “epic EHR” OR “epic EMR” OR “epic electronic health record*” OR “epic electronic medical record*”)

(“social determinants of health” OR SDOH OR “health social determinant*” OR “health structural determinant*” OR “structural determinants of health”) AND (“economic stability” OR “economic instability” OR income OR “income equality” OR “income inequality” OR “socioeconomic status” OR SES OR “socioeconomic factor*” OR poverty OR “financial resource strain” OR employment OR unemployment OR “basic needs”) AND (“electronic health record*” OR EHR OR “electronic medical record*” OR EMR OR epic OR “epic care” OR “epic EHR” OR “epic EMR” OR “epic electronic health record*” OR “epic electronic medical record*”)

(“social determinants of health” OR SDOH OR “health social determinant*” OR “health structural determinant*” OR “structural determinants of health”) AND (neighborhood* OR neighbourhood* OR communit* OR “built environment” OR “food insecurit*” OR “food rationing” OR “transportation needs” OR commuting OR “living arrangement*” OR “living situation” OR “food access” OR environment OR “environmental condition*” OR “housing stability”) AND (“electronic health record*” OR EHR OR “electronic medical record*” OR EMR OR epic OR “epic care” OR “epic EHR” OR “epic EMR” OR “epic electronic health record*” OR “epic electronic medical record*”)

(“social determinants of health” OR SDOH OR “health social determinant*” OR “health structural determinant*” OR “structural determinants of health”) AND (“health insurance” OR “insurance status” OR stress OR “psychological stress” OR depression OR “mental health” OR “mental illness” OR “mental disorder*” OR “emotional abuse” OR “psychosocial factor*” OR “psychological comorbidit*” OR “psychological abuse” OR education OR “health literacy”) AND (“electronic health record*” OR EHR OR “electronic medical record*” OR EMR OR epic OR “epic care” OR “epic EHR” OR “epic EMR” OR “epic electronic health record*” OR “epic electronic medical record*”)

(“social determinants of health” OR SDOH OR “health social determinant*” OR “health structural determinant*” OR “structural determinants of health”) AND (“social context” OR “social environment” OR “community context” OR “social connection*” OR “intimate partner violence” OR “intimate partner abuse”) AND (“electronic health record*” OR EHR OR “electronic medical record*” OR EMR OR epic OR “epic care” OR “epic EHR” OR “epic EMR” OR “epic electronic health record*” OR “epic electronic medical record*”)

### Suggested Search Strategy (MeSH/Keyword Combination) (MEDLINE & EMBASE)

1. exp "Social Determinants of Health"/

2. "social determinants of health".ab,ti.

3. SDOH.ab,ti.

4. "health social determinant* ".ab,ti.

5. "health structural determinant* ".ab,ti.

6. "structural determinants of health".ab,ti.

7. 1 or 2 or 3 or 4 or 5 or 6

8. "social risk factor* ".ab,ti.

9. "social factor* ".ab,ti.

10. exp Social Factors/

11. "behavioral factor* ".ab,ti.

12. "behavioural factor* ".ab,ti.

13. "life style".ab,ti.

14. "lifestyle factor* ".ab,ti.

15. exp Life Style/

16. exp Smoking/

17. exp "Tobacco Use"/

18. smoking.ab,ti.

19. "tobacco use".ab,ti.

20. "tobacco consumption".ab,ti.

21. "alcohol use".ab,ti.

22. "alcohol intake".ab,ti.

23. exp Alcohol Drinking/

24. exp Exercise/

25. "physical activity".ab,ti.

26. exercise.ab,ti.

27. "physical inactivity".ab,ti.

28. "inactiv*".ab,ti.

29. "lack of physical activity".ab,ti.

30. "sedentary behaviour".ab,ti.

31. "sedentary behavior".ab,ti.

32. sedentary.ab,ti.

33. "sedentary lifestyle".ab,ti.

34. "sedentary time".ab,ti.

35. exp Sedentary Behavior/

36. 16 or 17 or 18 or 19 or 20 or 21 or 22 or 23 or 24 or 25 or 26 or 27 or 28 or 29 or 30 or 31 or 32 or 33 or 34 or 35

37. "electronic health record* ".ab,ti.

38. EHR.ab,ti.

39. "electronic medical record* ".ab,ti.

40. EMR.ab,ti.

41. epic.ab,ti.

42. "epic care".ab,ti.

43. exp Electronic Health Records/

44. "behavior support".ab,ti.

45. "behaviour support".ab,ti.

46. "health behavior* ".ab,ti.

47. "health behaviour* ".ab,ti.

48. exp Health Behavior/

49. "behavioral health risk factor* ".ab,ti.

50. "behavioural health risk factor* ".ab,ti.

51. "behavioral health".ab,ti.

52. "behavioural health".ab,ti.

53. 8 or 9 or 10 or 11 or 12 or 13 or 14 or 15 or 44 or 45 or 46 or 47 or 48 or 49 or 50 or 51 or 52

54. "screening, brief intervention and referral".ab,ti.

55. SBIR.ab,ti.

56. SBIRT.ab,ti.

57. SBI.ab,ti.

58. "screening brief intervention referral to treatment".ab,ti.

59. 54 or 55 or 56 or 57 or 58

60. exp Economic Stability/

61. "economic stability".ab,ti.

62. "economic instability".ab,ti.

63. income.ab,ti.

64. exp Income/

65. "income equality".ab,ti.

66. "income inequality".ab,ti.

67. "socioeconomic status".ab,ti.

68. SES.ab,ti.

69. exp Social Class/

70. exp Socioeconomic Factors/

71. "socioeconomic factor* ".ab,ti.

72. poverty.ab,ti.

73. exp Poverty/

74. "financial resource strain".ab,ti.

75. employment.ab,ti.

76. exp Employment/

77. exp Unemployment/

78. unemployment.ab,ti.

79. "basic needs".ab,ti.

80. Economic Status/ or exp Poverty/ or (bankrupt* or "economic insufficiency" or ghetto* or impoverish* or insolven* or "lack of income" or "lack of money" or ((low or lower or lowest) adj3 (resourced or income*)) or "no income" or "no money" or ((poor or poorer or poorest) adj3 (household* or dweller* or income* or parent or parents or people or family or families or children or communit*)) or poverty or slum or slums or unemploy* or "low ses").mp.

81. 60 or 61 or 62 or 63 or 64 or 65 or 66 or 67 or 68 or 69 or 70 or 71 or 72 or 73 or 74 or 75 or 76 or 77 or 78 or 79 or 80

82. "neighborhood*".ab,ti.

83. "neighbourhood*".ab,ti.

84. "communit*".ab,ti.

85. exp Residence Characteristics/

86. "built environment".ab,ti.

87. exp Built Environment/

88. "food insecurit* ".ab,ti.

89. "food rationing".ab,ti.

90. exp Food Insecurity/

91. exp Transportation/

92. "transportation needs".ab,ti.

93. commuting.ab,ti.

94. "living arrangement* ".ab,ti.

95. "living situation".ab,ti.

96. "food access".ab,ti.

97. environment.ab,ti.

98. exp Environment/

99. "environmental condition* ".ab,ti.

100. "housing stability".ab,ti.

101. exp Food Supply/ or exp Gardening/ or exp Malnutrition/ or (hunting or fishing or gardening or "community garden*" or "community freezer*" or "growing vegetables" or traditional food* or "country food" or (wildlife adj3 harvest*) or (sustainab* adj3 harvest*) or malnourish* or undernourish* or "under nourish*" or underfed or ((nutrition* or dietary) adj3 (securit* or safety or insecurit* or inadequa* or adequa*)) or ((food or nutrition*) adj3 (policy or policies or subsid*)) or (food adj3 (accessib* or adequa* or availab* or unavailab* or desert or deserts or inadequa* or insecur* or local* or safety or sustainab* or scarcity or secur* or sharing* or supply or supplies or unsustainab*))).mp.

102. (exp *Transportation/ and (accessib* or afford* or availab* or barrier* or inaccessib* or inadequate or inconsisten* or poverty or reliab* or unafford* or unreliab*).ti,ab.) or ((transportation adj3 (accessib* or afford* or availab* or barrier* or inaccessib* or inadequate or inconsisten* or poverty or reliab* or unafford* or unreliab*)) or (lack* adj3 (transportation or vehicle* or driv* licen?e)) or "shut in" or "shut ins" or homebound or "home bound").mp. or Homebound Persons/

103. exp Homeless Persons/ or exp "Transients and Migrants"/ or ((vulnerable or migrant or transient*) adj2 (people or person* or individual* or child* or youth* or population* or worker* or men or women or man or woman)).mp. or (street adj2 (people or person* or individual* or youth* or population* or child* or men or women or man or woman)).mp. or ("lack of housing" or "hard to house" or "substandard housing" or "unstably housed" or underhoused or "under housed" or squatter* or homeless* or houseless* or unhoused or vagrant* or indigent or "couch surf*" or (sleeping adj3 rough) or "living rough" or "no fixed abode").mp. or (marginal* adj2 (population* or people* or group* or hous*)).mp.

104. 82 or 83 or 84 or 85 or 86 or 87 or 88 or 89 or 90 or 91 or 92 or 93 or 94 or 95 or 96 or 97 or 98 or 99 or 100 or 101 or 102 or 103

105. "health insurance".ab,ti.

106. exp Insurance, Health/

107. "insurance status".ab,ti.

108. stress.ab,ti.

109. "psychological stress".ab,ti.

110. exp Stress, Psychological/

111. exp Depression/

112. depression.ab,ti.

113. "mental health".ab,ti.

114. exp Mental Health/

115. "mental illness".ab,ti.

116. "mental disorder* ".ab,ti.

117. exp Mental Disorders/

118. exp Emotional Abuse/

119. "emotional abuse".ab,ti.

120. "psychosocial factor* ".ab,ti.

121. "psychological comorbidit* ".ab,ti.

122. "psychological abuse".ab,ti.

123. education.ab,ti.

124. exp Education/

125. exp Health Literacy/

126. "health literacy".ab,ti.

127. exp Educational Status/ or exp Student Dropouts/ or ((education* adj1 (status or achiev* or attain* or deficit or lack or level or levels or completion or completed or advanced)) or (diploma or "advanced degree" or schooling or "school leaver*" or "school drop out*" or "school dropout*" or "student drop out*" or "student dropout*" or uneducated or "poorly educated" or undereducated or "under educated" or graduates or "non graduate*" or nongraduate*)).mp. or (("high school" or postsecondary or "post secondary" or "highest grade") adj1 (achiev* or attain* or level or levels or completion or completed)).mp.

128. 105 or 106 or 107 or 108 or 109 or 110 or 111 or 112 or 113 or 114 or 115 or 116 or 117 or 118 or 119 or 120 or 121 or 122 or 123 or 124 or 125 or 126 or 127

129. "social context".ab,ti.

130. "social environment".ab,ti.

131. exp Social Environment/

132. "community context".ab,ti.

133. "social connection* ".ab,ti.

134. "intimate partner violence".ab,ti.

135. exp Intimate Partner Violence/

136. "intimate partner abuse".ab,ti.

137. exp Domestic Violence/ or exp Battered Women/ or exp Intimate Partner Violence/ or ipv.mp. or ((child or children or domestic or elder* or family or families or husband* or "inter generational*" or "intergenerational" or "intimate partner*" or sibling* or wife or wives or parent* or mother or mothers or father or fathers or grandparent* or grandfather* or grandmother* or uncle* or aunt*) adj3 (abuse or abused or abusive or batter* or beating* or beater* or trauma or violence)).mp.

138. 129 or 130 or 131 or 132 or 133 or 134 or 135 or 136 or 137

139. 16 or 17 or 18 or 19 or 20

140. 21 or 22 or 23

141. stair climbing/ or warm-up exercise/ or dancing/ or gardening/ or "play and playthings"/ or sports/ or exp athletic performance/ or exp martial arts/ or exp racquet sports/ or exp running/ or skating/ or exp snow sports/ or soccer/ or sports for persons with disabilities/ or exp swimming/ or "track and field"/ or volleyball/ or walking/ or weight lifting/ or wrestling/ or youth sports/ or exp Exercise/ or exp *Physical Education/ or (gymnasi* or green space* or "sports field*" or "play equipment" or "play* field*" or "movement break*" or "standing desk*" or "stand up desk*" or "standing workstation" or "stand up workstation*" or intramural* or playground* or recess or recreation* or sedentary or inactiv* or "outdoor facilit*" or exercis* or MVPA or vpa or mva or pedometer* or accelerometer* or fitbit* or aerobics or aikido or athletics or archery or badminton or ballet or bandy or barre or "base jumping" or basketball or biathlon or billiards or bobsleigh or bocce or "body building" or bouldering or boules or bowling or boxing or broomball or calesthentic* or cammag or camogie or "circuit training" or climbing or cricket or curling or cycling or dance or dances or dancing or discus or diving or fencing or football or futbol or gardening or golf or gymnastic* or handball or "hammer throw*" or "hang gliding" or "hip hop" or hockey or "horseback rid*" or hurling or javelin or jogging or judo or "jiu jitsu" or karate or kayaking or kickbox* or kiteboarding or "kung fu" or lacrosse or "lawn bowl*" or longboarding or luge or marathon* or "martial arts" or mountaineering or orienteering or paddling or parkour or pickleball or polo or powerlifting or qigong or racewalking or "racquet ball" or raquetball or ringette or rowing or rugby or running or sailing or shinty or skate or skateboarding or skating or snooker or snorkeling or snowshoe* or soccer or sport* or swim* or tai-kwan-do or taekwondo or "tai chi" or telemark or tennis or tobogganing or (track adj2 field) or triathlon or "ultimate frisbee" or ultramarathon* or volleyball or walking or weight lifting or weightlifting or windsurfing or wrestling or wushu or yoga).mp. or (((hallway* or play or classroom* or support* or bin or bins or break* or facilit* or space or spaces or resources or equipment) adj2 activ*) or (physical* adj2 (activ* or educat* or training or curricul* or literacy or compulsory or mandatory or daily)) or (activit* adj2 (track or record)) or (fitness adj2 (training or class* or program* or activit*))).mp.

142. 24 or 25 or 26 or 27 or 28 or 29 or 30 or 31 or 32 or 33 or 34 or 35 or 141

143. "epic EHR".ab,ti.

144. "epic EMR".ab,ti.

145. "epic electronic health record* ".ab,ti.

146. "epic electronic medical record* ".ab,ti.

147. 42 or 143 or 144 or 145 or 146

148. 37 or 38 or 39 or 40 or 41 or 43

149. 7 and 139 and 148

150. 7 and 140 and 148

151. 7 and 142 and 148

152. 7 and 53 and 148

153. 7 and 59 and 148

154. 7 and 81 and 148

155. 7 and 104 and 148

156. 7 and 128 and 148

157. 7 and 138 and 148

158. 147 or 149 or 150 or 151 or 152 or 153 or 154 or 155 or 156 or 157

159. 7 and 36 and 148

160. 158 or 159

161. limit 160 to english language

162. remove duplicates from 161
