# Supplementary material for: Integration of Screening and Referral Tools for Social Determinants of Health and Modifiable Lifestyle Factors in the Epic Electronic Health Record System: Scoping Review
Source: J Med Internet Res. 2025 Sep 15;27:e73615. doi: 10.2196/73615 (PMC12494108; doi:10.2196/73615)
Supplement: Multimedia Appendix 4 [file jmir_v27i1e73615_app4.docx]

Specific interventions including EHR integrations and strategies for screening in Epic for SDOH and risk factor domains and reported quantitative and qualitative outcomes

| **Author and year** | **Intervention** | **EPIC module or tools** | **Quantitative Results** | **Qualitative Results** |
| --- | --- | --- | --- | --- |
| **QUANTITATIVE OUTCOMES** | | | | |
| Angah 2024 [28] | Screening questionnaire added to pre-visit check-ins via MyChart. | MyChart | - Compared to baseline, ambulatory screening rates increased from 0.4% to 15.9% and inpatient screening rates increased from 0% to 66%.  - 10.7% of ambulatory patients and 15.7% inpatient patients screened and identified with at least one HRSN.  - Most prevalent HRSN was financial resource strain, then food insecurity. | N/A |
| Barclay 2019 [29] | Screening and counseling were implemented by using EPIC. | EPIC SmartPhrases, BPAs | - Of 9000 patients, 73.9% were screened and 39.7% were offered counseling after a positive screen.  - Nurses opened screening activity link in 62.6% of BPA firings | N/A |
| Brennan 2022 [31] | Depending on each practice, screening was completed either on paper or directly into EPIC. | Not reported | - Screening of eligible patients increased from 15% to 62%, discussions of screening results increased from 61% to 81%  - Fulfilled referrals increased from 37% to 57%, the rate of screening eligible patients quadrupled.  - Referring patients who screened positive remained relatively similar from 81% to 89%.  - Most improvements in screening and referrals were in the first 6 months of intervention. | N/A |
| Buitron de la Vega 2019 [10] | Screening was completed on paper then was inputted into EHR. The EHR then generates respective ICD-10 codes for referral. | Not reported | - 70% of new patients completed SDOH screening. - 26% of patients responded positively to at least 1 social need. Employment, food insecurity and problems affording medication were the most prevalent SDOH domains identified. - 22% of those who screened positive requested help, 86% of patients who requested resources received a relevant resource referral guide. - Medical assistant (MA) expended average ~1 minute extra to enter responses into EHR. | N/A |
| Bunce 2023 [32] | Short surveys were embedded into EHR for clinicians to complete 2 per day for 3 weeks. | Not reported | - SDOH was reported to influence 35% of surveyed encounters, 12% of surveyed encounters answered that SDOH had no influence on clinical decisions.  - SDOH were reported to influence care significantly more for males (vs females).  - Most common sources of patient SDOH information was conversations with patients, prior knowledge and the EHR. | N/A |
| Burdick 2017 [33] | A clinical decision support (CDS) tool was designed and implemented for screening to be done by staff and directly input into EHR. | BPAs | - The overall positive screen rate was 21% (179/866).  - Depression screening with PHQ-2 accounted for almost 60% of positive screens. Alcohol screening with AUDTI-1 represented 25%, while the prescription misuse question accounted for only 7% of the positive response.  - There were only 15 encounters (11%) with positive screens in 2 of the 3 domains, and no encounters included positive screens in all 3 domains.  - Screened patients were more likely to have behavioral health issues documented in past encounters compared with control patients who never received screening  - BH brief interventions and referrals were almost twice as frequent during encounters with screening compared to those without. | N/A |
| Cottrell 2019 [34] | Implemented EHR based screening tool. | Not reported | - 2% of patients visiting community health center were screened for SDOH (31,549 total), significant differences between screened vs. non-screened in sex (female), race (Hispanic/black), language, age, income (low), insurance status, homeless status (not homeless), migrant/seasonal status.  - 37,015 total screenings, increase in screenings per month throughout study. 50% of screening took place in 4 centers.  - Housing insecurity, relationship safety, and food insecurity most frequently documented of 7 SDOH domains.  - When SDOH-related help offered, 7% accepted, 21% declined, 71% did not answer. Help requested by patients in 2,246 screenings (6.1%) | N/A |
| Eakin 2023 [35] | Implementation of California State Bill 1152 (SB1152), homelessness screening, use of discharge Planning Guidelines, Checklists and Compliance. | Not reported | - The number of identified homeless patients doubled, with unique visits rising by 143%.  - Discharges increased from 73% to 81%, emphasizing better discharge processes.  - Admissions decreased by half (from 18% to 9%).  - 92% of identified patients had completed SB1152 discharge checklists.  - Visits by persons of the White race decreased from 50% to 40%, while visits by the Black, Asian, and Hispanic races increased from 18% to 25%, 1% to 4%, and 19% to 21%, respectively.  - Patients with only one ED visit decreased (28% to 22%) while those with four or more visits increased by 10% (46% to 56%). | N/A |
| Fiori 2019 [36] | Staff were trained in how to conduct screening and referrals. Screening was completed prior to medical visits via paper forms, which was later added into the EHR by a nurse. | Not reported | - 4948/6584 (72%) households were screened for SDOH with 984 (~20%) households reporting 1+ unmet social needs.  - 287/984 (~29%) of households received CHW assistance. 43% of households referred to CHWs had successful referrals. 49% of unsuccessful referrals were the result of the family being disconnected from CHW.  - The 3 most common referrals were housing stability and quality (40%), benefits assistance (19%), and food insecurity (15%). | N/A |
| Fiori 2020 [37] | Screening was done via paper forms at check-in and was then added into EHR. Referrals were made to community health workers within primary care centers in EHR. | Not reported | - Screening rate was 65% of the total visits (4162/6410).  - 19.7% (820/4162) had one or more positive responses. Among the positive SDOH screens, 37.8% (310/820) had more than one positive item.  - Three most common needs were childcare (48.8%), housing quality/availability (39.9%) and food (22.8%).  -The percent of “active” providers ranged from 50% to 100%, depending on the month, with an average of 76% of providers actively participating in screening over the study period. | N/A |
| Friedman 2018 [38] | SDH SmartSets in the patient navigator was used to identify and create referrals for a patient. | Not reported | - 11,273 patients with SDOH needs were identified and documented by patient navigators. The most identified SDH were inadequate material resources, needing assistance with community resources and financial problems  - Patient navigators made 18,284 unique community resource referrals (most common: transportation, government assistance programs) for 7494 patients (66%), with some patients receiving multiple referrals for different SDH needs. | N/A |
| Garg 2023 [39] | A WE CARE screening tool was used at check-in by patients themselves. This was then scanned into EHR where staff then used SmartPhrases in the AVS. | SmartPhrases | - Completed WE CARE screening ranged from 21%-43.8% across sites, and 28.9% of visits had the screener scanned into EHR.  - Over 20% of WE CARE families were referred to patient navigators, and the navigator connected with 73.4% of families. 27.7%-49.3% of parents who needed help received a referral.  - The most common referral was for childcare (25.8%), utilities (14.4%), and employment (14.1%).  - WE CARE families received significantly more resource referrals than control families (43.1% vs 1.9%). WE CARE children had significantly higher ED visits and hospitalization rates than control children | N/A |
| Gold 2023 [9] | A champion was identified among staff and was supported for 6 months. A dedicated trainer/coach met with clinic representatives 2-3 times a month for 3-6 hours. | Not reported | - The rate of SDOH screening increased by 2.45 times during intervention in comparison to pre-intervention. The impact of screening wasn't sustained post-intervention.  - Though screening was increased, no significant change was observed in referrals given despite increased patients requiring referrals. | N/A |
| Gore 2022 [41] | Screening was done based on USDA’s household food security survey in EPIC. When screened positive, nurses placed a consult order to social workers who provided a list of community resources. | Not reported | - 61% (361/587) patient encounters were screened and 5.8% identified as food insecure and were connected to a resource, compared to baseline screening rate of 2.2% before pilot project.  - There was an increase in screening seen from 14.3% then 44.4% (during 2-week pre-implementation nurse education period) reaching the peak of 82.1% during 3rd week of pilot project. | N/A |
| Gray 2023 [42] | Screening was completed on paper then transitioned to being done in EPIC in 2020. Only the data from EPIC was evaluated in the study. | Not reported | - 11,004/13,750 (80%) patients completed HRSN screening, 34.8% reported at least one social need. Of the patients that reported a social need, 79.2% received were offered resource information.  - A small portion (6.1%) of patients that reported one or more need were eligible for care navigation (they had 2 or more ED visits in the last year). 60.3% of eligible patients were offered care navigation services.  - Food insecurity was the most common social need. | N/A |
| Isaacs 2022 [48] | SDOH screening was completed by physicians during patient encounters. Resources were provided via an online link to social care providers. | Not reported | - 91.25% of pts (365/400) were screened for at least one SDOH category.  - 26.3% of screened pts had 1+ SDOH need (96/365). The most common identified SDOH need was food insecurity.  - 96 patients that screened positive received a follow up, 11 refused a referral. 6 additional patients refused assistance after contact with a health navigator. | N/A |
| Jennings 2022 [49] | Adults with cystic fibrosis were screened for SDOH. Patient's with MyChart access was sent a link for the online survey. Patients that did not do the screening received a reminder on their MyChart to complete the screening. If screening was done in the clinic, it was administered via paper instrument. | MyChart | - 132/142 patients (93%) completed the screening tool.  - 56/132 patients (42.4%) screened positive for SDOH needs.  - Only 4 patients requested assistance, but all 4 patients received follow up contact from a social worker and resources. | N/A |
| Gupta 2023 [45] | SDOH screening allowed patients to be directly connected to community resources through the EHR system. | NowPow | -Of 2687 patients screened, 662 (24.6%) positive for 1+ SDoH domains. Greatest SDOH need identified was food insecurity, financial strain, health literacy.  - 658/662 (24.5%) patients received SDoH referrals.  - The number of referrals received by patients decreased their primary care visits, but this effect was moderated by the number of comorbidities. | N/A |
| Jose 2020 [50] | Referrals were made to ambulatory oncology patients for tobacco use treatment through EPIC. | SmartPhrases, BPAs | - Out of the referred patients: 150 patients (71%) had a tobacco treatment appointment scheduled. 25 patients (17%) completed their tobacco treatment appointment.  - The study concluded that the EHR-based “opt-out” approach was feasible within the oncology clinical practice.  - Postimplementation: 4758 patients were seen, and 864 (18%) patients received a BPA. 210 of the patients were current tobacco users and referred to NDC by rooming staff. | N/A |
| Kroese 2024 [54] | A hunger vital sign questionnaire was conducted by physicians. Physicians were prompted to integrate smart phrases into patient notes and ICD-10 codes to track screening. Nurses often screened at the emergency department and clinics. | SDOH wheel and smart phrases | - Pediatric medical home screening increased from 0% to 80%.  - PED had median of 30% patients screened, which dropped to 16% when grant-funded gift cards were no longer available but increased back to 24% after implementation of SDOH wheel and EMR optimization.  - Specialty clinic screening increased to a median of 5%, further increasing to a median of 27% after distribution of results to food champions to each division.  - 9842 (20.9%) of patients were found to have food insecurity and provided resources.  - 895 (9%) families received food through primary community food pantry partner.  -398 (44%) families qualified for and were enrolled in USDA program to receive food. | N/A |
| Khanna 2021 [52] | Various strategies were utilized to implement an e-referral process including EPIC tip sheets, leadership buy-in, newsletters, training, educators, patient-focused advertisements, and distribution of video clips. | Not reported | - In 2018–2019, a total of 1,790 e-referrals were received by Quitline.  Among those referrals: - 18% accepted follow-up services and 18% declined the services.  - 64% were not reached after multiple attempts.  Of the 322 patients who accepted Quitline services:  - 55% requested nicotine replacement therapy.  - 282 clinicians referred patients, including 107 primary care physicians and 175 specialists.  - Notably, 62 clinicians emerged as “tobacco champions” by e-referring 72% of patients. | N/A |
| Lindenfeld 2023 [57] | N/A. Data was collected from EHR. | Not reported | - 17-21% of patients visiting large/mid-size facilities were screened for SDOH whereas only 7% of patients visiting small facilities were screened.  - In person visits at larger/mid-size facilities are more likely to conduct screening.  - Facilities with patients needing more SDOH needs will likely conduct more screening. | N/A |
| McCarthy 2021 [58] | The physical activity vital sign (PAVS) was embedded into Epic EHR. When patients checked in for their appointment, they were asked screening questions on electronic check-in kiosk. | Not reported | - Of the 1,322 patients, 72% (n = 951) completed the 3 PAVS questions from the single prompt at the check-in kiosk.  - 35% achieved at least 150 minutes of moderate or 75 minutes of vigorous PA per week.  - Significant factors associated with physical activity included sex, race, marital status, employment, body mass index and triglyceride levels.  - Those who had higher percentages of achieving PA recommendations were males; white; working full-time; overweight; and had lower triglycerides.  - Factors associated with not meeting current PA recommendations included being female, belonging to black or ‘other’ race categories, being retired or unemployed. | N/A |
| McNeely 2021 [59] | Screening was provided either through staff administration or self-administered (dependent on clinic resources and workflow). A brief counseling script was added into the EHR that could be used for patients with moderate to high-risk alcohol or drug use. | BPAsalerts | - 71.8% of eligible patients received screening for alcohol and 70.5% received screening for drugs.  - Screening rates were higher when screened for any visits vs annual examinations only.  - Counseling script used infrequently (rates of 0.1-12.5%). | N/A |
| Wang 2021 [68] | No specific intervention. Descriptive study on documentation of SDOH data. | Not reported | - Patient social history questions were rarely documented.  - Free-text patient social history fields had higher use.  - Documentation rates (% of hospital encounters): 0.03% social history questions, 0.09% problem list, 27.5% social work notes, 12.1% social history text, 86.0% inpatient nursing questions | N/A |
| LeLaurin 2023 [55] | From four pediatric clinics, parents were prompted to complete a social risk questionnaire and participated in qualitative interviews. | EPIC SDOH module | - Most participants identified as female, White, and non-Hispanic.  - Majority of families (70%) had multiple children. Parents lived in areas in the third and fourth social vulnerability index quartiles, indicating higher vulnerability.  - The parent sample had a higher proportion of individuals identifying as White (60% vs. 52%). A lower proportion identified as Hispanic (5% vs. 10%).  - 85% of the sample had one or more social risks. Risks fell under domains such as physical activity, safety, child education, food security, housing, caregiver health, and transportation.  - All parents scored ≥13 on the BRIEF assessment, indicating adequate health literacy. | N/A |
| Rogers 2022 [63] | Community resource network management software-as-a-service platform to identify and address SDOH. Screening of eligible patients was completed by self-screening, guardian screening (tablet or paper version), or by staff.  If positive for an SDOH, a referral was added to the after visit/discharge summary. | MyChart, BPA | - 111486 Medicare/Medicaid beneficiaries screened, 7878 SDOH identified, and 6103 beneficiaries were referred to community services.  - Incorporating BPAs that alert staff to screen for SDOH ensures patients are being screened.  - Having the AVS/discharge summary containing the personalized community resource services for each patient helps overcome literacy barriers. | N/A |
| Rudisill 2023 [64] | EHR embedded survey triggered input of community-based service information for the patient and located into the after-visit summary using NowPow/Unite Us. | NowPow | - 2.5% of visits (3630/147096) completed complete or partial SDOH screening.  - 38% (22/58) of practices completed screening.  - Physician assistants had greater odds of any SDOH screening than a medical doctor, nurse practitioners had significantly lower odds.  - Patients identified as Asian, Black or 2+ races were more likely to be screened compared to patients identified as White.  - Patients with managed care had greater screening odds compared to visits with patients that had private or commercial payers. | N/A |
| Sitapati 2020 [65] | Launched an enterprise-wide adoption of sexual orientation and gender identity documentation, built new electronic tools for clinical documentation and enabled web portal check in. Also built a depression registry for patients to identify their needs and have a follow up provided. Wellness, tobacco, hypertension, and diabetes registries were also added into the EHR. Primary care workflow was altered for tobacco status documentation, making it mandatory for back-office staff to ensure completion of tobacco screening | Not reported | - 17/18 sites exceeded performance targets for race, ethnicity, and language screening, 15/17 for sexual orientation and gender identity.  - Increased documentation of race/ethnicity/language from 27.1-94.5%, SO/GI from 0.15-71.3% | N/A |
| Stark 2024 [66] | Parents of patients were asked to complete the voluntary 12-item survey before or during the appointment.  If a patient had an active patient portal account, they received a screener 7 days before their appointment. Positive screens resulted in BPA for physician. | MyChart, BPA | - 1473/2046 (71.4%) patients completed screening through MyChart before or during their appointment.  - 472 (32%) patients screen positive for at least one SDOH domain, 48 (10.2%) patients who screened positive decline a referral. Financial strain, housing and food insecurity were the most prevalent concerns, respectively  -247/424 (58.3%) patients who screen positive received a referral and 230/424 (54.2%) had a documented encounter and received resources from a care member.  - 73.9% staff reported completing screening with patients and 70% reported reviewing patient chart for positive screenings. | N/A |
| Peretz 2023 [62] | Screening was done by the SDOH questions in EPIC. Ten new Patient Navigators were added to the workforce. Partnerships with community-based organizations were also formed to test and explore referral strategies. | Not reported | - 17.3% patients identified an SDOH need.  - Most common SDOH needs identified were housing, food, and transportation.  - Among rising/high risk identified patients, 50% accepted support and are working actively with the Patient Navigators (23% declined support).  - Including community-based organizations to develop a referral pilot program helped determine funds and capacity building for the project. | N/A |
| **QUALITATIVE OUTCOMES** | | | | |
| Grus 2021 [43] | Community health center (CHC) staff were interviewed about EHR-based SDOH screening without implementation support. | Not reported | N/A | - From the interviews, 3 key facilitators were identified to implement systematic SDH screening: external motivators, internal advocate who promoted screening and flexible approaches to develop screening workflows. |
| Palacio 2018 [60] | N/A - research team planned to collect SDOH data via MyChart patient portal and phone interviews. | MyChart | N/A | - Showing evidence of the impact of SDOH on outcomes did lead to buy-in.  - Regular meetings with leadership were key to deploying a road map for implementation.  - Important to bring together groups of stakeholders early on to clarify the expectations for the use of data and the preferred processes and workflows.  - A financial model that benefits from preventative strategies is key for the sustainability of the innovation. |
| **MIXED METHODS OUTCOMES** | | | | |
| Berkowitz 2021 [30] | Screening was completed during check in through EPIC, then a physician addressed social needs during appointment. | EPIC SDOH module | - 83% of pts responded to at least one SDOH question, responsiveness for 7/8 SDOH domains ranged from 55%-67%. Most identified SDOH was stress (33%).  - Visit lengths were 39.8 minutes which was 1.7 mins longer than pre-pilot visit lengths. | - In the survey, 90% believed SDOH information could help improve pt care/health and help improve therapeutic relationships with pts.- 1 in 3 responding physicians agreed having SDOH information would influence medical decision making. Limited awareness of SDOH resources to help confidently address SDOH needs. |
| Gold 2018 [40] | SDOH data collection and summary tools were deployed to CHCs. SDOH screening was done on paper, then was needed to put into EHR. | Not reported | - 97%-99% of the 1130 (n = 1098) screened patients had 1+ SDOH need.- Only 211 (19%) of the patients who had an SDOH need were given a referral. | - Staff required a lot of support when adopting SDOH EHR tools as new processes may interrupt their existing workflow. |
| Gunn 2023 [44] | Implemented 2-question social isolation and loneliness (SI/L) screening and EHR-integrated CRRP. | Not reported | - 963/4646 pts indicated feeling social isolation and loneliness, and 174 referrals were made.-9/15 pre-implementation interviews and 11/18 post-implementation interviews were completed. | - Participating CHCs reported being satisfied with their involvement in the pilot (increased knowledge, enabled prioritization of care). |
| Hao 2023 [46] | Patients identified by the research team were screened for SDOH via EHR. If needed, social work would contact the patient in clinic or by phone to provide resources. | SDOH wheel | - 112/137 eligible patients completed SDOH screening, with 107 patients having 1+ SDOH need. 20 patients were offered resources.  - Social connections was the most common identified need, followed by physical activity and tobacco). | - Interviewed patients (n=10) all shared a neutral or positive perspective on SDOH screening. Patients believe that providers should be aware of their social situations.  - Staff members found screening advantageous but saw difficulties in conducting screening due to interruptions and frustration from physicians. |
| Hsu 2018 [47] | Patients were referred to Community Resource Specialist (CRS) by staff, through EHR referral process or by self-referral. A patient's first visit with the CRS would be automatically entered in EHR-based CRS registry and CRS had tools to help work with patient. | Not reported | - 69% of referred patients had at least 1 complete visit documented, 11% had no CRS interaction but received resource information and 20% had no CRS interaction.  - Most frequent referral types were to social services, physical activity, support groups, health care resources and parenting support (45% of those referred patients reported using resource). | -In focus groups with pts, they reported behavior changes and improved health but no systematic difference in clinical/health status at population level was noticed.  - Integration of CRS role did not improve healthcare utilization at 3 or 6 months but improved experiences of other primary care team members. |
| Kepper 2023 [51] | The 10th revision of the International Classification of Diseases (ICD-10) allows for documentation of social needs in diagnostic and billing data system. Led to implementation Epic EHR to allow physicians to use ICD-10 codes for SDOH known as Z-codes. The study team pulled EHR data from 2015-2020 for z-codes during out-patient visits. Then healthcare providers and key stakeholders were recruited for semi-structured interviews on the dissemination and implementation of z-codes. | Not reported | - 1,019/118,251 (<1%) of patients had a documented social need.  -Within those who documented a social need, 14 (of 82) z-codes were used a total of 2147 times.  - Problems related to psychosocial circumstances accounted for 72.8% of codes and social environment was second and accounted for 12.3%. | -There was a lack of congruence between qualitative and quantitative findings in 5/7 SDOH categories.  - In interviews, 2 of the most recognized SDOH was housing/economic and education/literacy were not documented in z-codes. Similarly, the 2 most documented z-codes were not recognized as common patient needs during interviews. |
| Kostelanetz 2022 [53] | Physicians, advanced practice providers, outpatient nurses, social workers, case managers, pharmacists and administrators were anonymously surveyed via REDCap. Qualitative interviews were conducted with key stakeholders. | Not reported | -193/309 (62.5%) employees responded to the survey.  - 72% participants reported being somewhat familiar with SDOH screening.  - 93% of surveyors reported support for incorporation of social needs into healthcare and that screening should be part of standard care (89%).  - Healthcare professionals screened more routinely for health behaviors (alcohol, tobacco, drug use) than SDOH (housing, financial strain, food insecurity). | - An overwhelming number of survey participants reported social needs information could be used to improve patient care, communication and trust. |
| LeLaurin 2023 [56] | Clinics were using a third party SDOH questionnaire rather than the EPIC questionnaire. The screening was done before appointments via patient portal or on a tablet or paper at the clinic. | SDOH wheel | - Questionnaires scored highly (80-90%) for acceptability and appropriateness. | - Staff felt that module offered advantages over existing processes. Stated that domains were more comprehensive, and questions were more specific than existing questionnaires and screening procedures. SDOH wheel also helped provide a quick visual to assess risk factors. |
| Penedo 2022 [61] | Screening was completed prior to their appointment on MyChart which reminded the patient through email, portal, or phone call. An alert was sent to the medical team or social work (as appropriate) when a patient's screening was deemed necessary. Providers then followed up during clinic visits, through phone, or by MyChart messages. | MyChart, BPA | - 60% assessments were initiated, 70.2% of initiated were completed. 85.1% completed at home, 14.% completed at the clinic by staff.  - Majority of BPAs were for nutrition. Almost 100% of social workers' BPAs were addressed. | Four major themes:  - Most patients used portal and thought it was easy to navigate. - My Wellness Check program was useful if feedback/referrals were received by clinicians in a timely manner.  - Most patients preferred to complete assessments before appointments. - Identified lack of supportive care services during their cancer treatment as a major concern. |
| Wallace 2020 [67] | 10 questions from HealthLeads were chosen for screening as they felt these were best for the ED. Registration staff and/or clinical nurses screened patients. Linked REDCap and 211 & EPIC so that automated referrals would be made. Follow-ups for social needs were done by information specialists via phone. | Not reported | - 61% (129/210) patients screened with 1+ need with 52% (67) of screened patients wanting a follow up.  - 49% (32) of patients who wanted a follow up were contacted by 211 for referrals.  - Patients with social needs experienced a significant increase in ED use compared to those without needs (3 months post-ED visit). | - Staff sometimes felt uncomfortable with screening questions and used own discretion of which patients to screen. |
