# Supplementary material for: Integration of Screening and Referral Tools for Social Determinants of Health and Modifiable Lifestyle Factors in the Epic Electronic Health Record System: Scoping Review
Source: J Med Internet Res. 2025 Sep 15;27:e73615. doi: 10.2196/73615 (PMC12494108; doi:10.2196/73615)
Supplement: Multimedia Appendix 5 [file jmir_v27i1e73615_app5.docx]

Comprehensive table of study characteristics

| **Reference** | **Author and year** | **Location** | **Objective** | **SDOH Components** | **Setting** | **Population** | **Total (N)** | **Study type; Design** | **Intervention** | **EPIC Tools** |
| --- | --- | --- | --- | --- | --- | --- | --- | --- | --- | --- |
| **[1]** | Anagh 2024 | Connecticut, USA | To design, implement and increase standardized health related social needs (HRSN) screening and collection in EHRs across ambulatory and inpatient settings. | Food insecurity, transportation, financial strain, and housing insecurity. | Primary care and in-patient departments | Adults | 86079 | Quantitative; Prospective cohort | Screening questionnaire added to pre-visit check-ins via MyChart. | MyChart |
| **[2]** | Barclay 2019 | North Carolina, USA | To describe the implementation of screening and counseling for unhealthy alcohol use in primary care settings. | Alcohol use. | General internal medicine clinic | Adults | 9000 | Qualitative; Experimental (nonrandomized trial) | Screening and counseling were implemented by using EPIC. | EPIC SmartPhrases, Best practice advisories (BPA) |
| **[3]** | Berkowitz 2021 | California, USA | To screen and address SDOH, socioemotional development, and perinatal depression in pediatric practices through practice and system improvement. | Financial strain, transportation, stress, depression, violence, social connections, physical activity, and alcohol. | Ambulatory clinic | Adults 25-54 years old | 289 | Quantitative; Experimental (nonrandomized trial) | Screening was completed during check in through EPIC, then a physician addressed social needs during appointment. | EPIC SDOH module |
| **[4]** | Brennan 2022 | Indiana, USA | To screen and address SDOH, socioemotional development, and perinatal depression. | N/A | Pediatric practices | Children | 560 | Quantitative; Prospective cohort | Depending on each practice, screening was completed either on paper or directly into EPIC. | N/A |
| **[5]** | Buitron de la Vega 2019 | Massachusetts, USA | To understand social needs and test the feasibility of implementing SDOH screening supported by EHR. | Housing insecurity, food insecurity, medication affordability, transportation, utilities, care giving, employment, and education. | General internal medicine clinic | Adults | 1696 | Quantitative; Experimental (nonrandomized trial) | Screening was completed on paper then was inputted into EHR. The EHR then generates respective ICD-10 codes for referral. | N/A |
| **[6]** | Bunce 2023 | Oregon, USA | To explore how patient SDOH information influences and informs clinician decisions during clinical encounters. | N/A | Primary care clinics | Adults | 38 | Quantitative; Prospective cohort | Short surveys were embedded into EHR for clinicians to complete 2 per day for 3 weeks. | N/A |
| **[7]** | Burdick 2017 | Vermont, USA | To design and implement SBIRT using clinical decision support in EHR and evaluate the impact of tools on clinical outcomes. | Alcohol, depression, substance abuse. | Primary care practices | Adults (18-74) | 866 | Quantitative; Retrospective cohort | A clinical decision support (CDS) tool was designed and implemented for screening to be done by staff and directly input into EHR. | Best practice advisories (BPA) |
| **[8]** | Cottrell 2019 | USA (20 states) | To examine the adoption of SDOH screening in EHR. | Sex, race and ethnicity, language, age, income, insurance status, homelessness, and migrant status. | Community health centers | All | 31549 | Quantitative; Retrospective cohort | EHR based screening tool. | N/A |
| **[9]** | Eakin 2023 | California, USA | To assess the effects of SB1152 on ED visits by individuals experiencing homelessness.  To examine changes in demographics, clinical information, and repeat visits before and after SB1152 implementation. | Housing insecurity. | Stanford University Hospital | Adult (<18,  18-65, and >65 years) | 2160 | Mixed methods; Prospective cohort | The implementation of California State Bill 1152 (SB1152), homelessness screening, use of discharge Planning Guidelines, Checklists and Compliance. | N/A |
| **[10]** | Fiori 2019 | New York, USA | To develop and implement the Community Linkage to Care program and illustrate SDOH screening is feasible and practical. | Housing insecurity and quality, food, health care, utilities, transportation, and childcare and violence. | Ambulatory pediatric clinics | Children | 4162 | Mixed methods; Prospective cohort | Staff were trained in how to conduct screening and referrals. Screening was completed prior to medical visits via paper forms, which was later added into the EHR by a nurse. | N/A |
| **[11]** | Fiori 2020 | New York, USA | To assess the pilot integrating SDOH screening and referral in a primary care setting. Additionally, describe demographic and program factors associated with effectiveness. | Housing security, housing quality, benefits (such as utilities), food insecurity, transportation, medication, or health care access, childcare or eldercare assistance, legal services, relationship concerns, and safety issues. | Urban ambulatory pediatric clinics | Children | 4948 households | Quantitative; Prospective cohort | Screening was done via paper forms at check-in and was then added into EHR. Referrals were made to community health workers within primary care centers in EHR. | N/A |
| **[12]** | Friedman 2018 | Washington and Oregon, USA | To create and describe tools and processes used for developing strategies to address SDOH. | Social, economic, environmental, and health education. | Medical offices and hospitals | Adults | 11273 | Quantitative; Retrospective cohort | SDH SmartSets in the patient navigator was used to identify and create referrals for a patient. | N/A |
| **[13]** | Garg 2023 | Massachusetts, USA | To assess the implementation and effectiveness of the WE CARE social care system for low-income children. | Childcare, education, employment, food security, housing security, household heat, and language. | Community health centers | Parent-child dyads who presented for a newborn visit (child aged up until age 3). | 878 | Quantitative; Experimental (randomized trial) | A WE CARE screening tool was used at check-in by patients themselves. This was then scanned into EHR where staff then used smartphrases in the AVS. | Smartphrases |
| **[14]** | Gold 2018 | Oregon, USA | To assess the feasibility of implementing EHR tools for collecting, reviewing, and acting on SDOH data in CHCs. | Alcohol, race/ethnicity, tobacco use/exposure, depression, education, financial resource strain, housing insecurity, food insecurity, exposure to violence, physical inactivity, social isolation, and stress. | Community health centers | Adults (<65 years old) | 1130 | Mixed methods; Prospective cohort | SDOH data collection and summary tools were deployed to CHCs, while only 3 CHCs were using referral tools as they required customization. SDOH screening was done on paper, then was needed to put into EHR. | N/A |
| **[15]** | Gold 2023 | USA (8 states) | To examine if an implementation supported intervention improved EHR screening of social risks. | Child/family care insecurity, education, employment, financial strain, food insecurity, health insurance, health literacy, housing instability, inadequate physical activity, relationship safety, social isolation, stress, transportation needs, and utilities insecurity. | Community health center clinics | Adults | 531428 | Quantitative; Cross-sectional | A champion was identified among staff and was supported for 6 months. A dedicated trainer/coach met with clinic representatives 2-3 times a month for 3-6 hours. | N/A |
| **[16]** | Gore 2022 | New York, USA | To implement a sustainable process to screen hospitalized adults to capture individuals who would benefit from social work and food insecurity resources. | Food insecurity. | Hospital | Adults | 361 | Quantitative; Experimental (nonrandomized trial) | Screening was done based on USDA’s household food security survey in EPIC. When screened positive, nurses placed a consult order to social workers who provided a list of community resources. | N/A |
| **[17]** | Gray 2023 | Colorado, USA | To evaluate the implementation of HRSN screening and identify key lessons learned to inform expansion of HRSN screening and referral to other populations. | Housing insecurity, food insecurity, transportation, utilities, and safety. | Pediatric primary care clinic | Children (<19) | 11004 | Quantitative; Cross-sectional | Screening was completed on paper then transitioned to being done in EPIC in 2020. Only the data from EPIC was evaluated in the study. | N/A |
| **[18]** | Grus 2021 | USA (5 states) | To understand factors that facilitated introduction and integration of EHR based SDOH screening at CHCs. | Financial resource strain, food insecurity, housing insecurity, relationship safety, inadequate physical activity, social connection/isolation, and stress. | Community health centers | Adults | 43 staff | Qualitative; Experimental (randomized trial) | Community health center (CHC) staff were interviewed about EHR-based SDOH screening without implementation support. | N/A |
| **[19]** | Gunn 2023 | USA (Multistate) | To evaluate implementation of screening and community resource referral platforms (CRRPs) for social isolation and loneliness (SI/L) in CHCs. | Social isolation and loneliness. | Community health centers | Adults (50+) | 4646 patients and 18 staff | Mixed methods; Prospective cohort | Implemented 2-question social isolation and loneliness (SI/L) screening and EHR-integrated CRRP. | N/A |
| **[20]** | Gupta 2023 | South Carolina, USA | To assess the feasibility and sustainability of SDOH screening and referrals in a large health system, and how this affects health resources. | Food insecurity, housing instability, utility insecurity, transportation needs, financial instability, violence/abuse, language/educational needs, health literacy, social connectedness, and comorbidities. | Private nonprofit health  system, community health, in-patient  case management, or ambulatory care and condition management  programs | Adults | 2687 | Quantitative; Prospective cohort | SDOH screening allowed patients to be directly connected to community resources through the EHR system. | NowPow |
| **[21]** | Hao 2023 | North Carolina, USA | To evaluate the feasibility and acceptability of implementing an electronic health record SDOH screening instrument into routine, clinical, oncology practice. | Alcohol use, tobacco use, financial strain, food insecurity, transportation, social connections, physical activity, stress, housing, depression, and intimate partner violence. | American College of Surgeons Commission on Cancer accredited cancer center (outpatient clinic) | Adults with newly diagnosed gastrointestinal cancer (mean age 64.9) | 112 | Mixed methods; Prospective cohort | Patients identified by the research team were screened for SDOH via EHR. If needed, social work would contact the patient in clinic or by phone to provide resources. | SDOH wheel |
| **[22]** | Hsu 2018 | Washington, USA | To describe implementation of adding a lay health worker role into primary care and testing the feasibility and impact of the role. | N/A | Primary care clinics | Adults (18+) | 1182 | Mixed methods; Prospective cohort | Patients could be referred to Community Resource Specialist (CRS) by staff or through EHR referral process. Patients could also self-refer. A patient's first visit with the CRS, the patient would be automatically entered in EHR-based CRS registry and CRS had tools to help work with pt (intake, action planning and follow-up). | N/A |
| **[23]** | Isaacs 2022 | North Carolina, USA | To develop and implement SDOH screening at family care clinics where there was no previous standard of care. To generate awareness of SDOH occurrence, needs follow-up, and SDOH impacts on health outcomes. | Interpersonal violence, food security, financial strain, and transportation. | Primary care clinic | All | 256 adults (>18) and 144 children (<18) | Quantitative; Experimental (nonrandomized trial) | SDOH screening was completed by physicians during patient encounters. Resources were provided via an online link to social care providers. | N/A |
| **[24]** | Jennings 2022 | Virginia, USA | To reduce health disparities in individuals with cystic fibrosis by screening and addressing SDOH. | Housing insecurity, food insecurity, transportation, utilities, health-care access, medication access, income/employment, education. | Clinic | Adults | 142 | Quantitative; Experimental (nonrandomized trial) | Adults with cystic fibrosis who had at least one clinic encounter in 2021 were screened for SDOH. Patient's with MyChart access was sent a link for the online survey. Patients that did not do the screening received a reminder on their MyChart to complete the screening. If screening was done in the clinic, it was administered via paper instrument. | MyChart |
| **[25]** | Jose 2020 | Minnesota, USA | To enhance tobacco, use treatment among cancer patients by implementing an electronic health record (EHR)-based system for automatic referral to tobacco use treatment. | Tobacco use. | Comprehensive cancer center (Mayo clinic) | Not mentioned | 864 | Quantitative; Prospective cohort | Referrals were made to ambulatory oncology patients for tobacco use treatment through EPIC. | Smartphrases, Best practice advisories (BPA) |
| **[26]** | Kepper 2023 | Missouri, USA | To understand frequency of SDOH z-code use in EHR for patients with prediabetes and diabetes, explore factors that influence SDOH documentation in clinic care. | Employment, housing and economic circumstances, education and literacy, social environment, primary support group, problems related to upbringing, problems related to psychosocial circumstances. | Academic medical center | All, majority adults (2% under 18) | 118215 patients, 23 staff | Mixed methods; Cross-sectional | The 10th revision of the International Classification of Diseases (ICD-10) allows for documentation of social needs in diagnostic and billing data system. Led to implementation Epic EHR to allow physicians to use ICD-10 codes for SDOH known as Z-codes. The study team pulled EHR data from 2015-2020 for z-codes during out-patient visits. Then healthcare providers and key stakeholders were recruited for semi-structured interviews on the dissemination and implementation of z-codes. | N/A |
| **[27]** | Khanna 2021 | Maryland, USA | To develop an implementation strategy for electronic referrals to the tobacco Quitline within a large health system. | Tobacco use. | Ambulatory clinics | Adults | 1790 e-referrals (322 included in analysis) | Quantitative; Prospective cohort | Various strategies were utilized to implement an e-referral process including EPIC tip sheets, leadership buy-in, newsletters, training, educators, patient-focused advertisements, and distribution of video clips. | N/A |
| **[28]** | Kostelanetz 2022 | Tennessee, USA | To evaluate the perceptions that health care professionals hold of universal screening for SDOH. To investigate attitudes, facilitators barrier and perceived roles in implementation of screening for SDOH. | Housing status, social support, financial strain, food insecurity, educational attainment, alcohol use, tobacco use and drug use. | Academic medical center | Adults | 193 survey responders, 16 interviewees | Mixed methods; Cross-sectional | Physicians, advanced practice providers, outpatient nurses, social workers, case managers, pharmacists and administrators were anonymously surveyed via REDCap. Qualitative interviews were conducted with key stakeholders. | N/A |
| **[29]** | Kroese 2024 | Virginia, USA | To identify food insecurity and provide resources in real time to pediatric patients in need. | Food insecurity. | Pediatric clinics, emergency departments, and medical homes | Children | 9842 | Quantitative; Experimental (nonrandomized trial) | A hunger vital sign questionnaire was conducted by physicians. Physicians were prompted to integrate smart phrases into patient notes and ICD-10 codes to track screening. Nurses often screened at the emergency department and clinics. | SDOH wheel and smart phrases |
| **[30]** | LeLaurin 2023 | Florida, USA | To assess parent perspectives  on EHR-based social needs screening and documentation and  identify family-centered approaches for screening design and implementation. | Physical activity, food insecurity, housing insecurity, transportation needs, caregiver education and work, caregiver health, child education, and safety and environment. | Pediatric primary care clinics | All (Adults (age 31 to 56) and pediatric (>5 to <19)) | 20 parents | Qualitative; Cross-sectional | From four pediatric clinics, parents were prompted to complete a social risk questionnaire and participated in qualitative interviews. | EPIC SDOH module |
| **[31]** | LeLaurin 2023 | Florida, USA | To assess multi-level factors that may impact EHR-based social needs intervention adoption and develop stakeholder-informed intervention and implementation plans. | Not specified. | Pediatric primary care clinics | All | 48 patients and 30 staff | Mixed methods; Cross-sectional | Clinics were using a third party SDOH questionnaire rather than the EPIC questionnaire. The screening was done before appointments via patient portal or on a tablet or paper at the clinic. | SDOH wheel |
| **[32]** | Lindenfeld 2023 | New York, USA | To identify barriers to screening on a health system level and pinpoint organizational factors important to address to ensure that SDOH screening is comprehensively adopted across all clinic types. | Living situation, food insecurity, transportation, utilities, and safety. | Hospitals and community health centers | Adults | 542953 patients | Quantitative; Retrospective cohort | N/A. Data was collected from EHR. | N/A |
| **[33]** | McCarthy 2021 | New York, USA | To implement physical activity (PA) screening as part of the electronic kiosk check-in process in an adult preventive cardiology clinic and assess factors related to patients’ self-reported PA. | Physical activity. | Preventative cardiology clinic | Adults | 951 patients | Quantitative; Cross-sectional | The physical activity vital sign (PAVS) was embedded into Epic EHR. When patients checked in for their appointment, they were asked screening questions on electronic check-in kiosk. | N/A |
| **[34]** | McNeely 2021 | New York and Massachusetts, USA | To evaluate how to best implement EHR screening for substance use in primary care settings. | Alcohol and drug use. | Primary care clinics | Older adults (mean age 48-59) | 93114 | Quantitative; Experimental (nonrandomized trial) | Screening was provided either through staff administration or self-administered (dependent on clinic resources and workflow). A brief counseling script was added into the EHR that could be used for patients with moderate to high-risk alcohol or drug use. | Best practice advisories (BPA) alerts |
| **[35]** | Palacio 2018 | Florida, USA | To explore barriers and facilitators for integrating SDOH into the EHR system of a large, diverse healthcare system. | Physical activity, stress, housing insecurity, social connections, and medical access. | Hospitals and outpatient facilities | Adults | 37 stakeholders total (7 healthcare leaders, 4 IT staff, 5 physicians, 2 researchers, 4 healthcare staff, 10 patients, 2 regulatory/privacy roles) | Qualitative; Cross-sectional | N/A - research team planned to collect SDOH data via MyChart patient portal and phone interviews. | MyChart |
| **[36]** | Penedo 2022 | Florida, USA | To describe the feasibility and implementation of an electronic health record (EHR)–integrated symptom and needs screening and referral system in a diverse racial/ethnic patient population in ambulatory oncology. | Financial concerns, childcare, stress management, nutritional needs, emotional needs, transportation. | Ambulatory gynecology-oncology clinics | Adults | 506 patients | Mixed methods; Cross-sectional | Screening was completed prior to their appointment on MyChart which reminded the patient through email, portal, or phone call. An alert was sent to the medical team or social work (as appropriate) when a patient's screening was deemed necessary. Providers then followed up during clinic visits, through phone, or by MyChart messages. | MyChart, Best practice advisories (BPA) |
| **[37]** | Peretz 2023 | New York, USA | To describe an initiative that sought to identify and address underlying social needs. To describe experience of implementing systemwide planning and process for SDOH screening. | Housing insecurity, food insecurity, and transportation. | Emergency departments | Not mentioned. | 8318 patients | Qualitative; Experimental (nonrandomized trial) | Screening was done by the SDOH questions in EPIC. Ten new Patient Navigators were added to the workforce. Partnerships with community-based organizations were also formed to test and explore referral strategies. | N/A |
| **[38]** | Rogers 2022 | New Jersey, USA | To describe design, integration, and implementation of a systematic strategy to identify and screen inpatient, outpatient, and ED for SDOH utilizing EHR. | Housing instability, food insecurity, transportation problems, utilities, and interpersonal safety. | Hospital | Medicare/Medicaid beneficiaries (older adults (65+) who may have limited financial resources) | 111486 Medicare/Medicaid beneficiaries | Quantitative; Prospective cohort | They used a community resource network management software-as-a-service platform to identify and address SDOH. Screening of eligible patients was completed by self-screening, guardian screening (tablet or paper version), or by staff. If positive for an SDOH, a referral was added to the after visit/discharge summary. | MyChart, Best practice advisories (BPA) |
| **[39]** | Rudisill 2023 | South Carolina, USA | To identify barriers and facilitators of SDOH screening in primary care to inform future screening. | Food insecurity, financial strain, lack of transportation, stress, social isolation, violence/abuse, housing insecurity, exercise level of effort. | Primary care clinics | Adults | 78928 patients | Mixed methods; Cross-sectional | A 16-question EHR embedded survey was used for screening. Answers from SDOH screening then triggered input of community-based service information for the patient and located into the after-visit summary using NowPow/Unite Us. | NowPow |
| **[40]** | Sitapati 2020 | California, USA | To demonstrate how reliable care can be supported by standardized collection of SDOH data (race, ethnicity, language, sexual orientation, gender identity), stratification of quality measures, and EHR-based registries. | Race, ethnicity, language, sexual orientation, and gender identity. | Public hospitals | Adults | 17 hospitals, 35,000-41,000 patients depending on category | Quantitative; Prospective cohort | Launched an enterprise-wide adoption of sexual orientation and gender identity documentation, built new electronic tools for clinical documentation and enabled web portal check in. Also built a depression registry for patients to identify their needs and have a follow up provided. Wellness, tobacco, hypertension, and diabetes registries were also added into the EHR. Primary care workflow was altered for tobacco status documentation, making it mandatory for back-office staff to ensure completion of tobacco screening | N/A |
| **[41]** | Stark 2024 | Texas, USA | To assess implementation of new EHR-based SDOH screening and resource referral, and to evaluate the effectiveness and feasibility of implementing SDOH screening and referral. | Health literacy, transportation issues, food insecurity, housing stability, financial strain, and legal concerns. | Pediatric primary care clinic | Children | 1473 | Quantitative; Experimental (nonrandomized trial) | Parents of patients were asked to complete the voluntary 12-item survey before or during the appointment. If a patient had an active patient portal account, they received a screener 7 days before their appointment. Positive screens resulted in a best practice advisory sent to the physician in the EHR. | MyChart, Best practice advisories (BPA) |
| **[42]** | Wallace 2020 | Utah, USA | To develop and evaluate a process for systematically identifying social needs during routine health service delivery, for facilitating access to community-based supportive services, and for integrating existing clinical (i.e., Epic) and community-based referral data systems. | Housing insecurity, food assistance, transportation, mental health and addiction, employment, education, domestic violence, and abuse. | Emergency department | Adults | 210 | Mixed methods; Prospective cohort | 10 questions from HealthLeads were chosen for screening as they felt these were best for the ED. Registration staff and/or clinical nurses screened patients. Linked REDCap and 211 & EPIC so that automated referrals would be made. Follow-ups for social needs were done by information specialists via phone. | N/A |
| **[43]** | Wang 2021 | California, USA | To develop measures that capture use of SDOH screening: data capture and data review. | Transportation, financial strain, unstable housing, and food insecurity. | Hospitals and ambulatory clinics | Adults | 27127 patients | Quantitative; Retrospective cohort | N/A - data was collected from EHR | N/A |

*EHR – electronic health records; SDOH – social determinants of health; N/A - Not applicable

## References

1. Angah, N., et al., *Leveraging Technology and Workflow Optimization for Health-Related Social Needs Screening: An Improvement Project at a Large Health System.* Joint Commission Journal on Quality & Patient Safety, 2024. **50**(1): p. 24-33.

2. Barclay, C., et al., *Implementing Evidence-Based Screening and Counseling for Unhealthy Alcohol Use with Epic-Based Electronic Health Record Tools.* Joint Commission journal on quality and patient safety, 2019. **45**(8): p. 566-574.

3. Berkowitz, R.L., et al., *Evaluation of a social determinants of health screening questionnaire and workflow pilot within an adult ambulatory clinic.* BMC family practice, 2021. **22**(1): p. 256.

4. Brennan, L., et al., *Completion of Social Drivers of Health Screenings in*

*Pediatric Practices Participating in a Quality Improvement*

*Initiative.* Journal of Developmental & Behavioral Pediatrics, 2022. **43**.

5. Buitron de la Vega, P., et al., *Implementing an EHR-based Screening and Referral System to Address Social Determinants of Health in Primary Care.* Med Care, 2019. **57 Suppl 6 Suppl 2**: p. S133-s139.

6. Bunce, A., et al., *Patient-Reported Social Risks and Clinician Decision Making: Results of a Clinician Survey in Primary Care Community Health Centers.* Ann Fam Med, 2023. **21**(2): p. 143-150.

7. Burdick, T.E. and R.S. Kessler, *Development and use of a clinical decision support tool for behavioral health screening in primary care clinics.* Appl Clin Inform, 2017. **8**(2): p. 412-429.

8. Cottrell, E.K., et al., *Variation in Electronic Health Record Documentation of Social Determinants of Health Across a National Network of Community Health Centers.* American journal of preventive medicine, 2019. **57**(6 Suppl 1): p. S65-S73.

9. Eakin, M., et al., *Effects of California's New Patient Homelessness Screening and Discharge Care Law in an Emergency Department.* Cureus, 2023. **15**(2): p. e35534.

10. Fiori, K., et al., *From Policy Statement to Practice: Integrating Social Needs Screening and Referral Assistance With Community Health Workers in an Urban Academic Health Center.* J Prim Care Community Health, 2019. **10**: p. 2150132719899207.

11. Fiori, K.P., et al., *Integrating Social Needs Screening and Community Health Workers in Primary Care: The Community Linkage to Care Program.* Clin Pediatr (Phila), 2020. **59**(6): p. 547-556.

12. Friedman, N.L. and M.P. Banegas, *Toward Addressing Social Determinants of Health: A Health Care System Strategy.* The Permanente Journal, 2018. **22**(4S).

13. Garg, A., et al., *A Social Care System Implemented in*

*Pediatric Primary Care: A Cluster RCT.* Pediatrics, 2023. **152**.

14. Gold, R., et al., *Adoption of Social Determinants of Health EHR Tools by Community Health Centers.* Ann Fam Med, 2018. **16**(5): p. 399-407.

15. Gold, R., et al., *Implementation Support for a Social Risk Screening and Referral Process in Community Health Centers.* NEJM Catal Innov Care Deliv, 2023. **4**(4).

16. Gore, E., et al., *Implementing a Process for Screening Hospitalized Adults for Food Insecurity at a Tertiary Care Center.* Journal for healthcare quality : official publication of the National Association for Healthcare Quality, 2022. **44**(5): p. 305-312.

17. Gray, T.W., et al., *Examining the Implementation of Health-Related Social Need (HRSN) Screenings at a Pediatric Community Health Center.* J Prim Care Community Health, 2023. **14**: p. 21501319231171519.

18. Grus, I., et al., *Initiating and Implementing Social Determinants of Health Data Collection in Community Health Centers.* Population health management, 2021. **24**(1): p. 52-58.

19. Gunn, R., et al., *Implementation of Social Isolation Screening and an Integrated Community Resource Referral Platform.* J Am Board Fam Med, 2023. **36**(5): p. 803-816.

20. Gupta, D., et al., *Understanding the Role of a Technology and EMR-based Social Determinants of Health Screening Tool and Community-based Resource Connections in Health Care Resource Utilization.* Med Care, 2023. **61**(7): p. 423-430.

21. Hao, S.B., et al., *A Mixed-Methods Study to Evaluate the Feasibility and Acceptability of Implementing an Electronic Health Record Social Determinants of Health Screening Instrument into Routine Clinical Oncology Practice.* Annals of Surgical Oncology, 2023. **30**(12): p. 7299-7308.

22. Hsu, C., et al., *Evaluation of the Learning to Integrate Neighborhoods and Clinical Care Project: Findings from Implementing a New Lay Role into Primary Care Teams to Address Social Determinants of Health.* Perm J, 2018. **22**.

23. Isaacs, K., *Implementing Social Determinant of Health Screening in a Family Medicine Clinic: A Pilot Study.* Am J Med Qual, 2022. **37**(3): p. 200-206.

24. Jennings, D., et al., *Social determinants of health screening and intervention: A cystic fibrosis quality improvement process.* Pediatr Pulmonol, 2022. **57**(12): p. 3035-3043.

25. Jose, T., et al., *Design and Pilot Implementation of an Electronic Health Record-Based System to Automatically Refer Cancer Patients to Tobacco Use Treatment.* Int J Environ Res Public Health, 2020. **17**(11).

26. Kepper, M.M., et al., *The adoption of social determinants of health documentation in clinical settings.* Health Serv Res, 2023. **58**(1): p. 67-77.

27. Khanna, N., et al., *Electronic referrals to the tobacco Quitline: implementation strategies in a large health system to optimize delivery of tobacco cessation to patients.* Transl Behav Med, 2021. **11**(5): p. 1107-1114.

28. Kostelanetz, S., et al., *Health Care Professionals' Perspectives on Universal Screening of Social Determinants of Health: A Mixed-Methods Study.* Popul Health Manag, 2022. **25**(3): p. 367-374.

29. Kroese, L., et al., *Improving food insecurity screening across a health system throughout the COVID-19 pandemic.* BMJ Open Qual, 2024. **13**(1).

30. LeLaurin, J.H., et al., *Parent Perspectives on Electronic Health Record-Based Social Needs Screening and Documentation: A Qualitative Study.* Acad Pediatr, 2023. **23**(7): p. 1446-1453.

31. LeLaurin, J.H., et al., *Pediatric primary care provider and staff perspectives on the implementation of electronic health record-based social needs interventions: A mixed-methods study.* J Clin Transl Sci, 2023. **7**(1): p. e160.

32. Lindenfeld, Z., et al., *Assessing Differences in Social Determinants of Health Screening Rates in a Large, Urban Safety-Net Health System.* Journal of primary care & community health, 2023. **14**: p. 21501319231207713.

33. McCarthy, M.M., et al., *Implementing the physical activity vital sign in an academic preventive cardiology clinic.* Preventive medicine reports, 2021. **23**: p. 101435.

34. McNeely, J., et al., *Comparison of Methods for Alcohol and Drug Screening in Primary Care Clinics.* JAMA Netw Open, 2021. **4**(5): p. e2110721.

35. Palacio, A.M., et al., *Integrating Social Determinants of Health into the Electronic Health Records of a Large Health System: A Qualitative Perspective.* Perspectives in Health Information Management, 2018: p. 1-19.

36. Penedo, F.J., et al., *Implementation and Feasibility of an Electronic Health Record-Integrated Patient-Reported Outcomes Symptom and Needs Monitoring Pilot in Ambulatory Oncology.* JCO Oncol Pract, 2022. **18**(7): p. e1100-e1113.

37. Peretz, P., et al., *Social Determinants of Health Screening and Management: Lessons at a Large, Urban Academic Health System.* Jt Comm J Qual Patient Saf, 2023. **49**(6-7): p. 328-332.

38. Rogers, C.K., et al., *A Local Perspective into Electronic Health Record Design, Integration, and Implementation of Screening and Referral for Social Determinants of Health.* Perspect Health Inf Manag, 2022. **19**(Spring): p. 1g.

39. Rudisill, A.C., et al., *Patient and Care Team Perspectives on Social Determinants of Health Screening in Primary Care: A Qualitative Study.* JAMA Netw Open, 2023. **6**(11): p. e2345444.

40. Sitapati, A.M., et al., *A case study of the 1115 waiver using population health informatics to address disparities.* JAMIA open, 2020. **3**(2): p. 178-184.

41. Stark, K., et al., *Evaluation of a Clinic-Based, Electronic Social Determinants of Health Screening and Intervention in Primary Care Pediatrics.* Acad Pediatr, 2024. **24**(2): p. 302-308.

42. Wallace, A.S., et al., *Implementing a Social Determinants Screening and Referral Infrastructure During Routine Emergency Department Visits, Utah, 2017-2018.* Preventing chronic disease, 2020. **17**: p. E45.

43. Wang, M., et al., *Documentation and review of social determinants of health data in the EHR: measures and associated insights.* J Am Med Inform Assoc, 2021. **28**(12): p. 2608-2616.
